# Silsesquioxane Derivatives as Functional Additives for Preparation of Polyethylene-Based Composites: A Case of Trisilanol Melt-Condensation

**DOI:** 10.3390/polym12102269

**Published:** 2020-10-02

**Authors:** Dariusz Brząkalski, Robert E. Przekop, Bogna Sztorch, Paulina Jakubowska, Marek Jałbrzykowski, Bogdan Marciniec

**Affiliations:** 1 Faculty of Chemistry, Adam Mickiewicz University in Poznań, Uniwersytetu Poznańskiego 8, 61-614 Poznań, Poland; 2 Centre for Advanced Technologies, Adam Mickiewicz University in Poznań, Uniwersytetu Poznańskiego 10, 61-614 Poznań, Poland; robert.przekop@amu.edu.pl (R.E.P.); bs33013@amu.edu.pl (B.S.); 3 Faculty of Chemical Technology, Poznan University of Technology, Berdychowo 4, 60-965 Poznań, Poland; paulina.jakubowska@put.poznan.pl; 4 Faculty of Mechanical Engineering, Bialystok University of Technology, Wiejska 45 C, 15-351 Bialystok, Poland; m.jalbrzykowski@pb.edu.pl

**Keywords:** silsesquioxane, polyethylene, composite, nanocomposite, thermal degradation, processing, additive

## Abstract

In this work, polyethylene (PE) composites were prepared with a series of completely condensed silsesquioxanes (SSQ), as well as with open-cage hepta(*iso*butyl)trisilanol silsesquioxane. The effect of the additives on the thermal, mechanical, rheological, and crystalline properties of the composites obtained was determined. The dispersion of trisilanol derivative within polymer matrix was slightly better than that of the other *iso*butyl compounds, suggesting condensation of the additive to less polar products of different structure, which was confirmed by thermogravimetry (TG) and matrix-assisted laser desorption-ionization time-of-flight (MALDI-TOF) mass spectrometry analysis. The additives improved the thermal stability of polyethylene and formed composites of higher rigidity than the neat polyolefin. The results were compared to the literature data, with aminopropylhepta(*iso*butyl)silsesquioxane and vinylhepta(*iso*butyl)silsesquioxane being used partially as references, as PE composites thereof were reported earlier, but lacked some analytical results and required further investigation. It was proven that the practical upper loading limit for such silsesquioxane compounds as processing and functional additives for polyethylene should be fixed at around 1%.

## 1. Introduction

Polyolefins are among the most widely applied thermoplastic polymers as their reasonable price and tuneable properties, such as crystallinity level, melt-flow rate, or processing and service temperatures, render them highly versatile. Amongst them, polyethylene (PE) is by far the most commonly used. PE is utilized for production of foils, films, fibers, bags, tubes, containers, battery separators, and many more [1,2,3,4]. It is characterized by good thermal and electrical insulating properties, very low glass transition temperature (below −100 °C), high chemical inertness, high stress cracking resistance, and ease of processing. However, it has quite low mechanical properties, such as tensile strength, Young modulus or abrasion resistance, has limited thermal stability, very low heat deflection temperature, and poor flame resistance. To increase the number of possible applications of this polymer, different blends, copolymers, and grafted polymers thereof have been designed, as well as composites with numerous mineral and organic fillers or compositions with various processing and functional additives [5,6]. Fillers, such as natural fibers [4] or (nano)silica [7,8,9], have been applied to reinforce PE mechanically. To improve either processing or service properties of PE or composites thereof, additives such as lubricants, compatibilisers, plasticizers, or antioxidants/stabilizers have been extensively used [10]. As a novel type of either processing/functional additives or nanoreinforcing agents for polymer (nano)composites or blends, polyhedral oligomeric silsesquioxanes (SSQ) have been reported several times over the last twenty years, and discussed in detail in reviews by Kausar [11], Du [12], Kuo [13], Ayandele [14], Zhou [15], Shi [16], and Li [17]. Polyhedral oligomeric silsesquioxanes are known for their well-defined, molecular structure, controllable physicochemical properties on the basis of the introduced organic substituents, good solubility and dispersion properties, or simple, reproducible synthetic protocols [18,19]. As organosilicon hybrid compounds, SSQs tend to improve thermal stability of polymer matrices they are introduced into, but also modify materials rheology (e.g., as plasticizers or thixotropic agents) or improve mechanical properties, such as tensile or flexural modulus, tensile strength, heat deflection temperature. Different structures have been tested, including homo-(R_n_[SiO_1.5_]*_n_*) and heterosubstituted (R_n-m_ R’_m_[SiO_1.5_]*_n_*) systems, bearing either alkyl [20,21,22,23,24,25,26,27,28,29,30,31,32,33,34,35,36,37,38,39,40,41,42,43,44,45,46,47], alkenyl [41,48], aryl [24,25,26], or other functional groups [26,41,49,50,51,52], introduced mostly by condensation, hydrosilylation, or substitution reactions. It has been proven that a choice of introduced functional groups plays a major role in miscibility of silsesquioxanes and polymer matrix and the additive tendency towards agglomeration, but also the processing method and parameters have an impact on the obtained additive dispersion in the final composite or nanocomposite [40]. For example, large, linear alkyl chains render silsesquioxanes well-compatible with PE on the basis of similarity to the polyolefin chains, while smaller or branched substituents decrease the additive miscibility with the polymer [40,53]. At the same time, silsesquioxane additives of limited dispersion abilities may form particles serving as a reinforcing phase and improving mechanical properties of the obtained (nano)composite [21,25,40]. Also, it is important to point out that the processing method plays a huge role when discussing the preparation of SSQ/PE compositions. Very good dispersion of the additives has been obtained when high-shearing-forces processes have been applied, such as twin-screw extrusion [22,23,44] or injection moulding [32]. Among other methods, ball milling [30,31,33,37], solution blending [34,35], or reactive melt blending [51,52] have been reported with moderate to good results. The dispersion level plays a huge factor on the properties of the obtained materials, and effective dispersing of the organosilicon additive is crucial to observe effects such as (nano)reinforcing action or thermal stabilization of the composition [11,12,13,14,15]. In the previous work, we showed how a proper choice of organic substituents for spherosilicate compounds, as well as a processing method applied, allowed for effective obtaining of micro-and nanodispersion of the compounds in PE together with limited agglomeration [54]. It is crucial to mention that often two levels of dispersion can be observed and above specific concentration (depending on the compound and polymer matrix) multi-micron agglomerates are formed, together with a fraction of the additive being well-dispersed at a sub-micron scale [55]. In this work, a critical assessment of simple silsesquioxanes application as functional additives for PE was made, including compatibility of the components with the polyolefin matrix and the effects associated with increasing additive loading. For this purpose, a series of silsesquioxanes was applied as such additives, and their dispersion ability and impact on the obtained composite systems were studied. Melting and crystallization, thermal stability, mechanical and basic rheological properties were investigated. Additionally, the behaviour of open-cage hepta(isobutyl)trisilanol silsesquioxane, *i*Bu_7_SSQ-3OH, under processing temperature was investigated. To the best of our knowledge, besides of one attempt [38], it was not studied as a direct additive for polyethylene or polypropylene, probably due to concerns about its polarity rendering the compound immiscible with highly apolar polyolefin.

## 2. Materials and Methods

### 2.1. Materials and Instrumentation

The chemicals were purchased from the following sources: *iso*butyltrimethoxysilane, trichlorovinylsilane, chloropropyltrichlorosilane, chloropropyltriethoxysilane, aminopropyltrimethoxysilane from ABCR, triethylamine and chloroform-d from Sigma Aldrich, tetrahydrofuran (THF), methanol, hydrochloric acid, acetonitrile and acetone from Avantor Performance Materials (Poland). Low density polyethylene (LDPE) type Malen E FABS 23 D022 from BasellOrlen Polyolefins. Its characteristics are *M*_w_ = 66,000, MFR (190 °C; 2.16 kg) = 2 g/10 min, *T*_m_ = 112 °C and density 0.921 g/cm^3^. Silsesquioxanes were prepared according to the literature procedures [56,57,58,59]. *i*Bu_7_SSQ-Vi was synthesised according to the procedure for the synthesis of *i*Bu_7_SSQ-Cl. SSQ-8Cl was obtained as a resinous cage mix and was used as such.

Polyethylene concentrates were prepared on a two-roll mill Zamak Mercator WG 150/280. The concentrates were ground in SHINI SG-1417 low-speed mill and dried for 6 h at 90 °C. Prepared concentrates were then diluted to final additive concentrations in an extrusion process with cold granulation using Thermo Fisher Scientific (Waltham, MA, USA) HAAKE PolyLAB OS extrusion setup equipped with a single screw (D = 19.05 mm, *L*/*D*: 25) working at 30 rpm. The temperature zones for extrusion were set, from feed to die, as follows: 120, 160, 180, and 180 °C. For foils production, the setup was equipped with a foil extrusion die and chill-roll system. The foils prepared were of 0.2 mm thickness. Dumbbells were prepared on ENGEL E-Victory 170/80 injection moulding machine equipped with a double socket mould for preparation of normalized testing specimens consistent with the PN-EN ISO 527-2 norm (type 1A). The parameters for injection moulding were as follows: temperature profile of plastification unit, from feed to die: 170, 180 and 190 °C; mould temperature: 22 °C; injection time: 2 s; cooling time: 20 s; injection pressure p1: 1200 bar, p2: 500 bar; holding pressure profile: 1200 bar/1 s, 1000 bar/4 s, 300 bar/5 s.

MFR index was measured on INSTRON CEAST MF20 plastometer equipped with 2.16 kg weight at temperature of 190 °C.

For tensile strength tests, a universal testing machine Instron 5969 was used, in accordance to the norm EN ISO 527-2: 1996. The speed of traverse was set to 50 mm/min for dumbbells and 100 mm/min for foils.

^1^H, ^13^C, and ^29^Si nuclear magnetic resonance (NMR) spectra were recorded at 25 °C on a Bruker Ascend 400 and Ultra Shield 300 spectrometers using CDCl_3_ as a solvent. Chemical shifts are reported in ppm with reference to the residual solvent (CHCl_3_) peaks for ^1^H and ^13^C.

Fourier transform-infrared (FT-IR) spectra were recorded on a Nicolet is 50 fourier transform spectrophotometer (Thermo Fisher Scientific) equipped with a diamond ATR unit with a resolution of 0.09 cm^−1^.

SEM/EDS analyses were recorded on a Quanta FEG 250 (FEI) instrument; SEM at 5 kV and EDS at 30 kV, respectively. The samples were frozen in liquid nitrogen and fractured with pliers to reveal a surface satisfactory for an analysis.

Thermogravimetry was performed using NETZSCH 209 F1 Libra gravimetric analyser. Samples of 5.0 ± 0.2 mg were cut from each granulate and placed in Al_2_O_3_ crucibles. Measurements were performed under nitrogen and air in a 30–800 °C temperature range and at 20 °C/min temperature rise. Differential scanning calorimetry was performed using NETZSCH 204 F1 phoenix calorimeter. Samples of 5.0 ± 0.2 mg were cut from each granulate and placed in aluminium crucible with punched lid. Measurements were done under nitrogen in 20–220 °C temperature range and at 10 °C/min temperature rise. Each sample was treated by two heat-cool cycles to erase its thermal history and the data presented were measured for the second cycle. Crystallinity was determined from the first melting. For determination of materials crystallinity, fusion enthalpy of 100% crystalline PE of 288 J/g was assumed, according to literature reports [60].

### 2.2. General Procedure for Composites Preparation and Characterization

In a typical procedure, about 200 g of polyethylene was rolled on a two-roll mill until complete melt, after which the chosen modifier was added in a quantity corresponding to 5% of the final composite content, and the composition was rolled together at temperature range of 180–185 °C until it became completely homogeneous or until no more improvement of homogeneity was observed. After that, the composition was taken off the rolls and set to cool down. It was ground in the low-speed mill and the obtained masterbatch granulate was then diluted to 1.5%, 1%, and 0.5% by mixing it with the granulate of neat PE in a proper proportion and extruding it on a single-screw extruder, and the extrudate being simultaneously granulated. 0.1% concentration composites were obtained by diluting 0.5% granulate. For *i*Bu_7_SSQ-3OH, satisfactory dispersion was observed at 0.5% and therefore 0.1% concentration composite was not prepared. On the other hand, *i*Bu_7_SSQ-Vi was found to be the most prone to form agglomerates and therefore further diluting it to 0.1% concentration was abandoned. 1.5% concentrations were prepared only with *i*Bu_7_SSQ-3OH and *i*Bu_7_SSQ-Vi, to compare the behaviour of two highly different additives at high loading: poorly miscible, crystalline *i*Bu_7_SSQ-Vi, and well-miscible *i*Bu_7_SSQ-3OH forming amorphous condensation products. The obtained granulates were then measured by SEM, TG, and DSC techniques, and processed into standard dumbbell specimens and foils. Foils were cut into proper specimens using a sample cutter, equipped with a knife in a form of a dumbbell. Tensile strength tests for dumbbells and foil specimens were performed to measure ultimate tensile strength and elongation at the point of plastic elongation limit, as well as Young’s modulus.

## 3. Results and Discussion

### 3.1. Characterisation of the Obtained Modifiers

**Figure 1:**
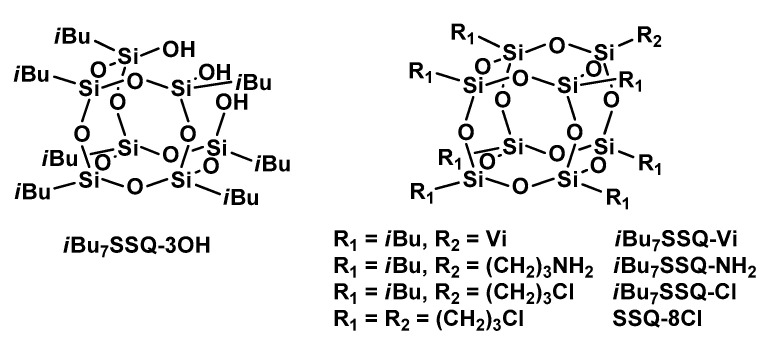
The Silsesquioxane (SSQ) compounds used in this work.

The silsesquioxanes used in this work are presented on Figure 1.

**Figure 2:**
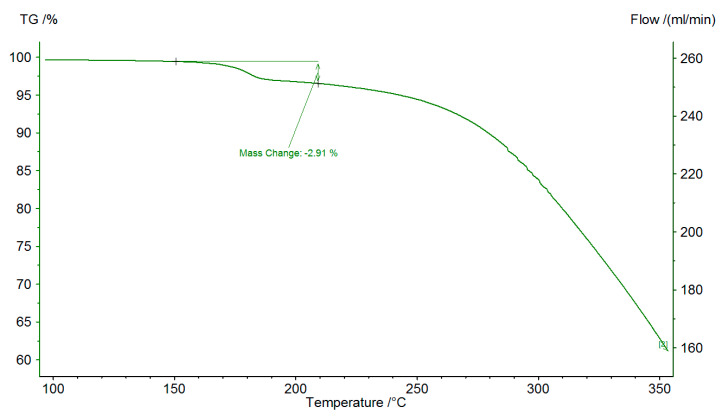
TG analysis of *i*Bu_7_SSQ-3OH showing initial mass loss (cropped for visibility).

**Scheme 1:**
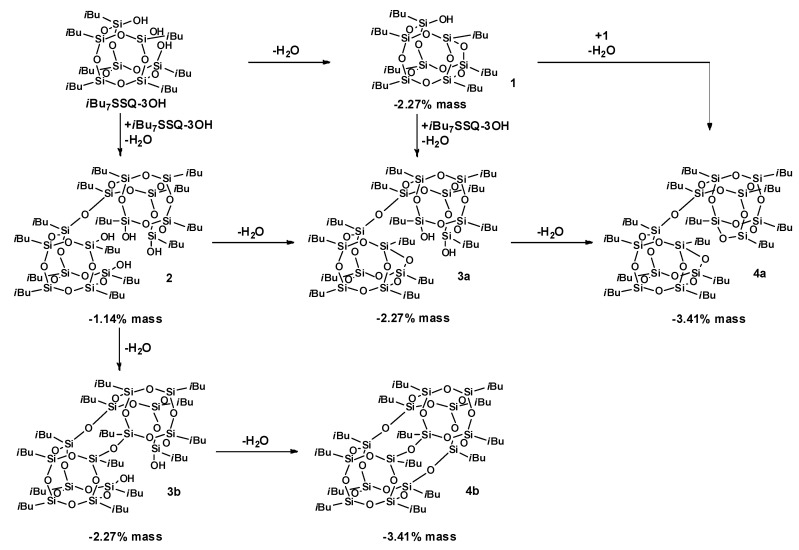
Possible routes of thermal condensation of hepta(isobutyl)trisilanol silsesquioxane. Depending on the condensation product, mass loss is presented in accordance to either one (1) or two molecules (2, 3a, 3b, 4a, 4b) of the initial trisilanol.

The structure of all the modifiers presented was confirmed spectroscopically (NMR, FT-IR, see Table S1 and Section 2 in the Supplementary Materials). TG analysis of the compounds allowed for verification of thermal stability of most of the additives under the conditions of PE processing, as well as reactivity of *i*Bu_7_SSQ-3OH, and suggested some of the mechanisms of their thermal degradation at elevated temperatures. For SSQ-8Cl, the product was obtained in two fractions of different form: crystalline, in less than 15% yield, and amorphous in over 65% yield. The crystalline product was found to be pure T_8_ cage, but the yields were not satisfactory for further use. The TG of the amorphous product did not show any mass loss under 300 °C, and ^29^Si NMR did not confirm presence of any free silanol groups (see Figure S4 in the Supplementary Materials), which confirmed full condensation, however the product was an amorphous mixture of different cage hybrids (cage mixture) and Figure 1 represents the idealized structure. The amorphous product was chosen for the composite preparation. For *i*Bu_7_SSQ-3OH, an unusual two-step mass loss was observed, with the first degradation event occurring at a relatively low temperature. The mass loss of ~3% observed in the 150–210 °C temperature range, presented in Figure 2 (DTG peak at 180.3 °C), during heating of hepta(isobutyl)trisilanol suggested dehydrative condensation of the compound with the formation of a mixture being a combination of some idealized products presented in Scheme 1, as well as some higher molecular weight products of the cage rearrangement and further condensation.

When a sample of *i*Bu_7_SSQ-3OH was heat-treated in a laboratory dryer for 6 min at 180 °C (the time required for full sample melt), it turned into a glassy, transparent material. MALDI-TOF-MS analysis of heat-treated samples allowed for identification of all the proposed low-molecular products, that is a monomer and dimers (see Scheme 1) according to the molecular weight of the molecule (see Figures S17 and S18 in the Supplementary Materials), but no attempt was made to isolate them and identify their precise molecular structures. Therefore, condensation products bearing the same molecular weight were marked with the same numbers (e.g., Scheme 1. 3a,3b). Similar conclusions on degradation and condensation of trisilanol were drawn by Zeng et al. when heat-treating hepta(*iso*octyl)trisilanol, however MALDI-TOF mass spectra didn’t allow for identification of any particular product [61]. It is possible that, during thermal treatment, *i*Bu_7_SSQ-3OH undergoes condensation to products of higher molecular weight than the ones presented, however those would exceed *m/z* ratio limit available for the instrument used. More findings in the subject of MALDI-TOF-MS analysis of silsesquioxanes and silsesquioxane-derived materials may be found in some recent scientific reports [62,63,64].

### 3.2. SEM-EDS Analysis

**Figure 3:**
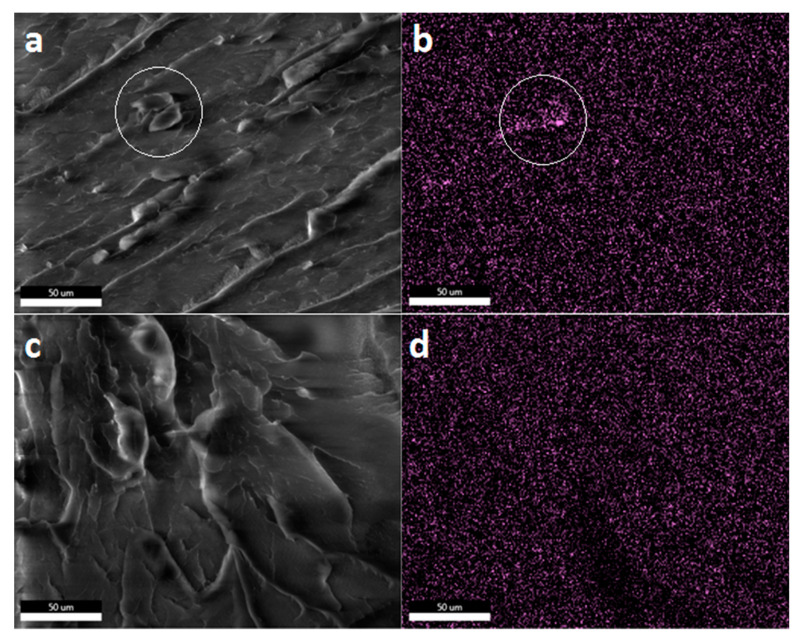
SEM images (**left**) and EDS maps of silicon (**right**) of 1% *i*Bu_7_SSQ-3OH/PE composite obtained by extrusion (**a**,**b**) and injection moulding (**c**,**d**).

SEM imaging combined with EDS mapping of silicon allowed for assessment of additives dispersion within PE. For the assessment of silsesquioxane compounds dispersion abilities, the SEM imaging of all the studied materials was first performed on the SSQ/PE extrudates obtained. The dispersion of SSQ-8Cl was satisfactory at all concentrations, showing good compatibility of the non-polar, amorphous additive with the polymer matrix. Also, *i*Bu_7_SSQ-3OH showed comparably good compatibility with PE, which can be explained on the basis of TG and MALDI-TOF-MS analyses, by the additive forming less polar condensation products upon mixing with molten polymer. Only at 1.5% loading, substantial agglomeration was observed. For 1% *i*Bu_7_SSQ-3OH/PE, on the basis of SEM and EDS, some agglomerates were observed for the samples prepared by extrusion (Figure 3a,b), but no agglomerates were visible for the injection-moulded specimens in the scale applied for this study (Figure 3c,d). This proves that the additional shearing forces occurring during injection moulding (which is a high temperature, high pressure process) improve the dispersion of the additive. At 1.5%, some agglomeration was observable even in injection-moulded samples, showing the limitation of the additive dispersion abilities. In comparison, the other additives, all bearing *iso*butyl groups, showed dispersion properties similar to each other, but inferior to the ones discussed above. In all cases, at higher loadings agglomerates can be observed forming, which is easily visible on EDS maps (see Figures S18–S35 in the Supplementary Materials). The most significant self-aggregation was observed for *i*Bu_7_SSQ-Vi, where substantial agglomerates were already observable at 0.5% loading, and at 1.5% very only a small fraction of the additive remains highly dispersed. This presents the limited compatibility of *iso*butyl-substituted and other small alkyl group-substituted silsesquioxanes with polyethylene under different processing techniques, especially with their increasing loading, which was previously presented by Guo and Fréchette [30,31,33,34,35,43,44,45]. Additionally, the presence of a different substituent in one corner of hepta(*iso*butylsil)sesquioxanes may introduce dipole moment, increasing polarity of the molecule and reducing the compatibility of the compound with nonpolar PE chains. This may be the reason for better compatibility of products of *i*Bu_7_SSQ-3OH thermal treatment during composite processing in comparison to the other *i*Bu_7_SSQ derivatives used in this work. Also, the amorphous state of *i*Bu_7_SSQ-3OH heat-treated samples, as well as mix-cage SSQ-8Cl, both being mixtures of various condensation products, may play a role in the dispersion mechanism, contrary to *i*Bu_7_SSQ-Vi, *i*Bu_7_SSQ-NH_2_ and *i*Bu_7_SSQ-Cl, all being crystalline solids, which may promote their self-aggregation, as speculated by Sheen [65] and Perrin [66]. Also, Grala et al. reported on segregation of *i*Bu_7_SSQ-NH_2_ additive during crystallization of HDPE composites, as well as two different levels of dispersion, where the size of additive particles varied from less than 100 nm up to several microns [51].

### 3.3. Mechanical Analysis

Mechanical studies have been performed to evaluate mechanical properties of the obtained materials. The results are collected in Table 1, Table 2 and Table 3. Some different observations were made upon analysis of two groups of specimens: dumbbells and foils. It was obvious that the form of product the polymer material is made into and especially the processing technology used to obtain the given object have a huge impact on the final properties of the objects and some compounds, as much useful as processing additives for one type of processing technology, may be inappropriate for another one. Very similar observations were made when studying spherosilicate-derived PE processing additives [54]. For dumbbells, in most cases a small increase in tensile strength was observed, with a general trend of strength improvement being smaller for higher concentrations, most likely due to high additive dispersion at lower loadings, as it was proven with SEM-EDS. This small increase can be linked to the presence of silsesquioxane particles in the polymer, forming a reinforcing phase. Agglomerates forming at higher loadings no longer serve reinforcing action, and rather introduce mechanical defects in the materials. Similar observations were made by Lim et al. [25]. However, differences were subtle and often within the limits of standard deviation, probably due to overall low loadings of the additives tested, the silsesquioxane component making up a small fraction of the composition volume. Some higher values of tensile strength were obtained for *i*Bu_7_SSQ-NH_2_ and *i*Bu_7_SSQ-3OH, therefore better compatibility between the PE and particles of mentioned silsesquioxanes may be speculated. All these observations suggest good uniformity of the materials, obtained by high-temperature, high-shearing forces process that injection moulding is. At the same time, it presents limited utility of applied compounds as functional additives when speaking of mechanical properties improvement. Hato et al. showed how the addition of octamethylsilsesquioxane, known for its tendency of forming agglomerates within PE, decreased mechanical properties, that is tensile strength and elongation at rupture, when applied at 5–10% loadings (lower loadings were not tested) [47]. At such a concentration, the additive only increased Young’s modulus, as is typical for a classic solid filler, e.g., silica [7,8,9]. Grala et al. reported the addition of *i*Bu_7_SSQ-NH_2_ to generally impart the mechanical parameters of HDPE composites both at static load and on impact, as must have been a result of high additive loading and low additive-polymer interaction [51]. Better results were obtained for HDPE-*g*-MA and *i*Bu_7_SSQ-NH_2_ reactive blending experiments, however it is difficult to compare such results with those on physical compounding, as reactive blending is chemical modification of the polymer, resulting in changes on the molecular level of the polymer chain. Also, grafted polymers, such as HDPE-*g*-MA, already show properties different than those of neat homopolymers, and are far more expensive, usually intended for use in small quantities as compatibilizing agents for polymer blends or composites with mineral fillers.

SSQ-8Cl and *i*Bu_7_SSQ-3OH showed trends of increasing Young’s modulus of the composites, the well-dispersed additives probably increasing chain aggregation in the amorphous phase of the polymer and reducing free volume. For the other additives, the effect of material stiffness increase can also be seen, most likely due to additive particles mechanically reinforcing the composite, but at higher loadings the effect decreased as the particle agglomerates introduced too many discontinuities in the material. As most of the systems showed improved material rigidity, it resulted in small reduction of plastic elongation values for all samples, which is usually expectable in terms of comparison between neat polymers and composites derived thereof (see Table 3).

For the foil samples, for almost all examples, a rather severe reduction of tensile strength was observed, which can be linked to particles of additive disturbing linear flow and orientation of polymer in the foil. Together with reduced Young’s modulus and plastic elongation, it represents deterioration of foils properties. *i*Bu_7_SSQ-3OH/PE system, however, showed similar behaviour of tensile strength change for 0.5% loading, but at 1.0% it surprisingly exceeded tensile strength of the neat polymer, similarly to the dumbbell specimens (Table 1). Together with relatively high Young’s modulus (comparable to neat PE foils) and severely decreased value of plastic elongation, it suggests a complex interaction between PE, the highly dispersed products of *i*Bu_7_SSQ-3OH condensation, and less dispersed particles thereof, acting as a reinforcing phase, despite the general tendency of silsesquioxane particles deteriorating foil properties. Therefore, the *i*Bu_7_SSQ-3OH composite foils presented satisfactory rigidity, but not exceeding that of neat PE within standard deviation (Table 3). At 1.5% loading, tensile strength of *i*Bu_7_SSQ-3OH/PE foils remained high, but due to increased additive agglomeration, the material showed heavily decreased values of Young’s modulus and plastic elongation. Similar to the dumbbell specimens, all the foils presented decreased plastic elongation, however the change was more severe. The results are, in general, similar to the previously reported observations on spherosilicate/PE composites and prove that such cage siloxane systems are a rather poor choice for additives for PE foils processing [54].

### 3.4. Thermomechanical Analysis

**Figure 4:**
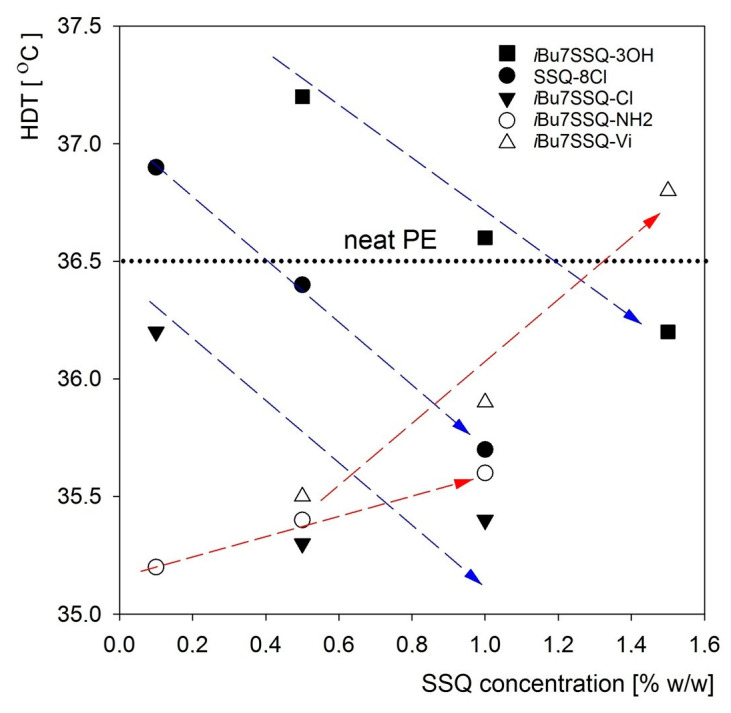
Heat Deflection Temperatures of the obtained composites.

Heat deflection temperature (HDT) analysis was performed to analyse effect of the SSQ additives on the samples’ stiffness under conditions of increasing temperature (Figure 4, Table S3 in the Supplementary Materials). Some interesting trends were observed. SSQ-8Cl and *i*Bu_7_SSQ-3OH initially caused increase of the HDT values, which corresponds to higher sample stiffness under given temperature. However, with increased loadings, the amorphous additives behaved like plasticizing agents, decreasing HDT values below that of the neat PE. The effect was especially prominent for SSQ-8Cl at 1.0% loading. *i*Bu_7_SSQ-Cl also showed plasticizing character, but the effect was visible already at 0.1% loading. It was likely caused by the additive particles causing small disorders of the polymer matrix. On the other hand, *i*Bu_7_SSQ-NH_2_ and *i*Bu_7_SSQ-Vi initially decreased HDT values of the composites thereof, but with increasing loading, the parameter also increased. The initial HDT decrease may be explained the same as for the *i*Bu_7_SSQ-Cl, by the additive particles introducing disorders of the polymer matrix, however, at higher concentrations, the particles of the crystalline additive most likely provide mechanical reinforcement of the SSQ/PE composite, rendering the composite more stiff, as these compounds do not undergo any softening or melting in the temperature range applied for this study.

### 3.5. Thermal Analysis

Thermogravimetric analysis was performed to study the impact of the applied organosilicon additives on the thermal stability of the obtained materials. The results are collected in Table 4. In general, all the obtained compositions showed improved thermal stability, however degradation mechanisms differ in air and ambient atmosphere (nitrogen), and the stabilising effect of silsesquioxanes on the polymer matrix was more significant in air, therefore it will be studied in detail. The nonlinearity of the stabilising action may be linked to two factors, as neither of them allows for full explanation of the observed behaviour on its own. One cause for this effect is the dispersion/agglomeration described above, as it impacts basically all the properties studied for these materials. The second effect is the critical concentration at which the additive moderates a degradation mechanism and above which no further change can be expected (the saturation effect), e.g., free radical reactions of the polymer chain during polyolefin cracking, where only a small concentration of free radicals is present at the time. It should be also noted that even at the polymer melting temperature, simple silsesquioxane additives with small substituents (*i*Bu, Vi, substituted propyl) should not be expected to dissolve or melt, and rather only disperse/agglomerate, besides *i*Bu_7_SSQ-3OH during the initial compounding process. The above general conclusions are similar to the previous findings on spherosilicate/PE composites [54]. It can be speculated that the siloxane framework of the silsesquioxane molecule plays a role in a recombination or quenching of free radicals, as an average Si–C bond is weaker than a C–C bond, which results in elimination of an organic group and formation of a silyl radical [67]. Similar conclusions were given by Fina et al. upon studying thermal degradation of various octasilsesquioxanes [68]. For most of the additives, the *T*_5%_ parameter decreased upon the increase of additive concentration from 0.5% to 1%, where additive agglomeration was observed, as it translates to decreased concentration of species available towards reaction with free radicals on cracking PE chains. This effect was less severe for *T*_onset_ and *T*_DTG_, probably due to additive further dispersing or reacting with the cracking polyolefin at high temperature, as may be in case of *i*Bu_7_SSQ-3OH. At 1.5% loading, the additive showed basically the same results to those at 0.5%, as the increased amount of the compound likely compensated for the agglomeration effect. At 0.5% concentration, the SSQ-8Cl/PE composition showed some of the highest thermal parameters, representing good stabilising effect of the additive. The presence of chlorine atom in the structure may play the role in the mechanism of composition stabilization, as it is known that chlorinated compounds are susceptive towards recombination with silyl radicals, as well as other radical species [69]. However, the most prominent thermal stabilising agent was *i*Bu_7_SSQ-NH_2_, where together with increasing loading, a rise of all thermal parameters was observed. This effect can be explained by the presence of amino group, which serves as a free radical sweeper and a reducing agent, as well as metal deactivator of polyolefin polymerization catalyst leftovers, which also play a role of a catalyst of high temperature polyolefin degradation in oxidative atmosphere [10,70]. Nguyen et al. described the reactivity of aminoalkylated silsesquioxane towards recombination with peroxyl radicals formed on PE chains, however a more complex composition was described, where together with iron(III) stearate, an amine-mediated catalytic oxidation system was formed [49]. Grala et al. reported a significant increase of *T*_5%_ parameter for *i*Bu_7_SSQ-NH_2_/HDPE composition, however *T*_DTG_ parameter was unaffected [51]. Similarities between thermal behaviour of the remaining SSQ additives described in this work are due to their similarities of chemical structure, which was also observed while studying dispersion and crystallinity. Interestingly, Bouza et al. reported a decrease of *T*_5%_ and *T*_onset_ upon the addition of 2% *i*Bu_7_SSQ-NH_2_ and a small increase of *T*_onset_ upon 10% addition, when studying composites thereof with isotactic polypropylene, however the measurements were only done in argon atmosphere [71].

DSC analysis shows that all the additives affect melting and crystallisation temperatures of the obtained compositions (see Table 5). When discussing crystallisation temperature (*T*_c_), the most important role of the additives to consider is the nucleating effect, observable by increased *T*_c_ values. In general, all the additives exhibited nucleating effect. For SSQ-8Cl, a trend for increasing nucleating effect associated with increasing additive loading was visible. For *i*Bu_7_SSQ-Cl, the nonlinearity was most likely caused by the additive agglomeration at 0.5% loading, and additional nucleating effect of highly abundant agglomerated particles observed by 1% loading. The strongest effect was recorded for *i*Bu_7_SSQ-NH_2_, however a similar agglomeration effect was observed by 0.5% concentration. *i*Bu_7_SSQ-Vi was moderately effective, with nucleating effect decreasing together with increasing loading, and *i*Bu_7_SSQ-3OH showing an opposite trend. Barczewski et al. reported a significant increase of crystallization temperature of LDPE characterized by low nominal *T*_c_ (96.3 °C) and also observed only a minor difference between 0.5% and 1% loading, showing the “saturation effect” of the increasing additive concentration [41].

The changes of the melting temperature can be linked to moderation of mean crystallite size, and larger crystallites being responsible for higher melting temperature [54]. By this principle, all the compounds mediated the PE crystallites size, with a tendency for most additives to reduce the main crystallite size at the highest additive loading. As the amount of additive increases, additional particles of non-melting silsesquioxane compound provide more nucleation sites, and the high number of simultaneously growing spherulites limit their size due to small volume available for growth of each spherulite. An opposite trend was observed for *i*Bu_7_SSQ-Vi, most likely due to the additive showing strong agglomeration tendency, therefore providing less nucleation sites together with increasing loading. Also, SSQ-8Cl at 0.1% loading and *i*Bu_7_SSQ-3OH at 0.5% and 1.0% loadings effectively induced growth of crystallites larger than those of neat PE. This is likely due to low concentration of nucleating species, initiating a non-disturbed spherulite growth. Grala et al. reported little impact of *i*Bu_7_SSQ-NH_2_ on melting and crystallization temperatures of HDPE composites at 2.5% and 5% loading, which showed the limited functionality of such additives at higher concentrations, where increased segregation occurs [51].

**Figure 5:**
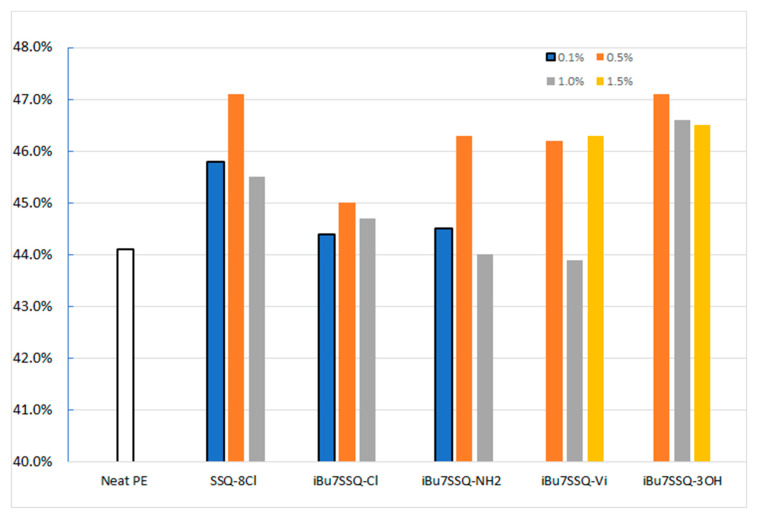
Crystallinity indices (CI) of the obtained composites.

Additionally, DSC was used to determine the crystallinity indices (CI) of the obtained materials. In general, very small effect of the additives on crystallinity levels of polyethylene in obtained composites was observed (Figure 5, Table S2 in the Supplementary Materials), however, in all cases, the nucleating effect of the additives was confirmed, as the CI values are above those of neat PE. Joshi et al. showed that addition of octamethylsilsesquioxane to HDPE at various loadings had little impact on crystallinity level of the polymer, however it mediated its crystallite size [23]. Guo et al. also suggested that when applying octa(*iso*butyl)silsesquioxane for UHMWPE, the additive segregated on the spherulite interfaces, where most of the polymer amorphous phase is located [37]. The non-linear relationship between the SSQ additive loading and the crystallinity levels of PE can be explained on the basis of two levels of dispersion of the additives, where at higher loadings more agglomerates form and the less numerous, large particles of the additive provide less nucleation sites for the crystallizing polymer [54,55]. For most examples, the highest crystallinity can be observed at 0.5% loading, so it can be speculated that it’s the concentration where most of the additive is well dispersed and provides the highest amount of small, nucleation-promoting particles, whereas at 1.0% and 1.5% loadings, multi-micron agglomerates predominate, limiting the number of available nucleation sites. The exact relationship between the additive concentration and the nucleating effect of the additive would be dependent on the type of functional groups present in the compound structure, and a matter of more detailed study, which was not covered in this work.

### 3.6. Rheological Analysis

Basic rheological analysis was performed by assessing the effect of silsesquioxane additives on melt flow rate of the obtained compositions (Table 6). In all cases the additive caused reduction of MFR value, however there was an interesting general trend for this reduction to decline and the MFR values closing to that of the neat polymer together with increasing loading. It suggests that the additives disrupt the flow of composition, either adding thixotropic character to the composition in their particulate form (which is a normal behaviour for molten polymers containing particulate fillers), or by increasing polymer chains interaction, when dispersed on a molecular level. However, with increasing concentration, the additional modifier worked as a lubricating agent, likely by reducing adhesion between the composition and barrel, similarly to silicone additives, which is a positive feature in a perspective of application of such compositions for injection moulding and extrusion technologies. For *i*Bu_7_SSQ-Vi, an increase of MFR index was observed together with increasing loading, up to 2.03 value at 1.5% concentration, which is comparable to that of the neat PE. Similarly, Barczewski et al. reported that the addition of *i*Bu_7_SSQ-Vi to LDPE considerably reduced kinetic viscosity of the compositions at low shear rates, with a trend for further viscosity reduction together with an increasing amount of the additive used [41].

## 4. Conclusions

Most of the compounds investigated in this work showed tendency for agglomeration at higher loadings, and the most effective concentration was usually such where the additive was highly dispersed without high accompanying agglomeration. The compositions with 1.5% loading were similar in characteristics to those of 0.5% or worse. It is due to limited compatibility between PE and silsesquioxanes bearing small organic substituents. Therefore, for PE processing, it should be considered not to exceed loadings of around 1% of such compounds, especially taking into account the production cost of these compounds. Hepta(isobutyl)trisilanol silsesquioxane was highly reactive at PE processing temperature, resulting in condensation into less polar, amorphous species showing good compatibility with PE, better than that of the investigated close-caged silsesquioxanes, which translated into good mechanical and thermal properties of the obtained compositions. Similar behaviour was observed for SSQ-8Cl/PE composites, the highly symmetrical and apolar, as well as amorphous additive being well-miscible with the polymer matrix. Also, *i*Bu_7_SSQ-NH_2_ exhibited very good thermal stabilizing effect in oxidative atmosphere. The processing method type had a great impact on the mechanical properties of the materials obtained, as due to high shear forces applied, the injection moulded SSQ/PE samples were characterised by much better performance than the extruded foils. The method provided better homogenisation of the polymer with the additives, as confirmed by SEM-EDS. Nevertheless, the SSQ compounds had a limited impact on the mechanical properties of the obtained composites, as contrary to different literature reports. However, it was mainly due to low loadings of the additives applied, as the higher concentrations were not considered in this work. All the investigated compounds showed a limited nucleation effect on PE and moderated the mean crystallite size of the polymer. Also, at higher loadings, SSQ-8Cl and *i*Bu_7_SSQ-3OH served as plasticizing agents.

In the light of the presented results, as well as other reports, it should be pointed out that the use of the term “nanofiller” to describe polyhedral oligomeric silsesquioxanes is inadequate. Polyhedral oligomeric silsesquioxanes may be applied as functional additives (either thermal, mechanical, anti-oxidative, or processing, e.g., rheological, crystallization-mediating), but due to their multifunctionality, and also relatively high costs, they cannot be considered fillers, as opposed to silica or other affordable mineral or organic materials being applied for this purpose to either reduce production costs or considerably improve mechanical properties of the composite obtained. Probably, this misinterpretation was formed due to the initial fascination over the unique cage structure of polyhedral oligomeric silsesquioxanes, where another inaccurate name for these systems was introduced, i.e., molecular silica. Moreover, for polyethylene systems, at higher loadings, they tend to agglomerate up to a degree where most of the particles or agglomerates cannot be considered nanoscopic anymore.

## Figures and Tables

**Table 1:** Tensile strength of the SSQ/PE composites.

Additive	Sample	Concentration of Additive (%)			
		0.1	0.5	1.0	1.5
		Tensile Strength (MPa)			
Neat PE	Dumbbell	13.08 ± 0.26			
	Foil	19.70 ± 0.95			
SSQ-8Cl	Dumbbell	13.11 ± 0.33	13.29 ± 0.19	13.22 ± 0.34	-
	Foil	16.79 ± 0.77	15.99 ± 0.88	15.87 ± 0.81	-
*i*Bu_7_SSQ-Cl	Dumbbell	13.41 ± 0.12	13.29 ± 0.21	13.14 ± 0.27	-
	Foil	14.44 ± 0.69	16.44 ± 0.43	15.45 ± 0.71	-
*i*Bu_7_SSQ-NH_2_	Dumbbell	14.43 ± 0.22	14.20 ± 0.49	14.06 ± 0.19	-
	Foil	18.17 ± 0.39	16.42 ± 0.90	16.62 ± 0.77	-
*i*Bu_7_SSQ-Vi	Dumbbell	-	13.00 ± 0.34	12.98 ± 0.32	12.52 ± 0.48
	Foil	-	16.31 ± 0.94	14.49 ± 0.86	14.91 ± 0.80
*i*Bu_7_SSQ-3OH	Dumbbell	-	14.41 ± 0.24	14.36 ± 0.18	14.33 ± 0.32
	Foil	-	15.83 ± 0.68	20.81 ± 0.80	19.12 ± 0.70

**Table 2:** Young’s modulus of the SSQ/PE composites.

Additive	Sample	Concentration of Additive (%)			
		0.1	0.5	1.0	1.5
		Young’s Modulus (MPa)			
Neat PE	Dumbbell	91.68 ± 3.46			
	Foil	213.82 ± 14.59			
SSQ-8Cl	Dumbbell	92.92 ± 5.31	95.93 ± 4.71	97.71 ± 5.39	-
	Foil	166.08 ± 8.70	155.35 ± 6.13	164.17 ± 9.64	-
*i*Bu_7_SSQ-Cl	Dumbbell	94.34 ± 3.08	95.86 ± 4.24	92.73 ± 2.01	-
	Foil	150.74 ± 8.08	168.26 ± 8.74	141.10 ± 7.47	-
*i*Bu_7_SSQ-NH_2_	Dumbbell	108.92 ± 2.70	101.80 ± 4.87	103.75 ± 4.77	-
	Foil	209.89 ± 11.23	159.42 ± 6.48	166.67 ± 8.39	-
*i*Bu_7_SSQ-Vi	Dumbbell	-	92.53 ± 4.84	92.08 ± 3.55	98.63 ± 4.46
	Foil	-	151.69 ± 9.03	166.20 ± 9.40	150.88 ± 11.05
*i*Bu_7_SSQ-3OH	Dumbbell	-	109.09 ± 2.08	109.72 ± 3.40	105.67 ± 2.82
	Foil	-	167.58 ± 7.14	194.06 ± 9.79	110.74 ± 6.35

**Table 3:** Plastic elongation of the SSQ/PE composites.

Additive	Sample	Concentration of Additive (%)			
		0.1	0.5	1.0	1.5
		Elongation at Maximum Load (%)			
Neat PE	Dumbbell	60.38 ± 1.77			
	Foil	176.69 ± 11.63			
SSQ-8Cl	Dumbbell	59.97 ± 2.84	59.25 ± 2.15	57.97 ± 3.41	-
	Foil	166.05 ± 7.83	142.70 ± 6.30	158.96 ± 9.29	-
*i*Bu_7_SSQ-Cl	Dumbbell	60.29 ± 2.14	59.45 ± 2.06	59.86 ± 1.15	-
	Foil	121.55 ± 6.92	112.41 ± 5.33	101.40 ± 5.23	-
*i*Bu_7_SSQ-NH_2_	Dumbbell	57.69 ±1.18	59.28 ± 2.52	58.03 ± 2.02	-
	Foil	161.07 ± 6.13	159.16 ± 7.11	137.63 ± 5.77	-
*i*Bu_7_SSQ-Vi	Dumbbell	-	59.05 ±2.02	59.28 ± 3.01	53.51 ± 1.32
	Foil	-	158.60 ± 8.02	141.78 ± 8.28	133.73 ± 9.06
*i*Bu_7_SSQ-3OH	Dumbbell	-	57.13 ± 0.76	56.52 ± 1.28	57.88 ± 0.90
	Foil	-	113.53 ± 5.41	124.40 ± 6.13	76.24 ± 4.12

**Table 4:** Thermal parameters of SSQ/PE composites from TGA measurements.

**Measurements in Air Atmosphere**							
**Additive Conc. [%]**	***T* (°C)**	**Additive Type**					
		**Neat PE**	**SSQ-8Cl**	**iBu_7_SSQ-Cl**	**iBu_7_SSQ-NH_2_**	**iBu_7_SSQ-Vi**	**iBu_7_SSQ-3OH**
0.1	*T*_5%_	348.3	355.4	355.6	362	-	-
	*T*_onset_	382.4	379.4	380.2	400.3	-	-
	*T*_DTG_	418.3	422.6	419.8	448.5	-	-
0.5	*T*_5%_	348.3	373.3	362.3	365.5	366.6	364.7
	*T*_onset_	382.4	399.4	391.7	434.7	398.5	396.9
	*T*_DTG_	418.3	455.8	440.8	445.4	443.9	436.5
1.0	*T*_5%_	348.3	362.5	354.4	381.4	359.9	369.0
	*T*_onset_	382.4	392.1	394.3	448.4	389.7	404.6
	*T*_DTG_	418.3	439.1	433.7	458.1	447.2	438.5
1.5	*T*_5%_	348.3	-	-	-	364.7	372.8
	*T*_onset_	382.4	-	-	-	402.5	404.7
	*T*_DTG_	418.3	-	-	-	444.4	441.9
**Measurements in Nitrogen Atmosphere**							
**Additive Conc. [%]**	***T* (°C)**	**Additive Type**					
		**Neat PE**	**SSQ-8Cl**	**iBu_7_SSQ-Cl**	**iBu_7_SSQ-NH_2_**	**iBu_7_SSQ-Vi**	**iBu_7_SSQ-3OH**
0.1	*T*_5%_	441.8	447.4	446.5	444.9	-	-
	*T*_onset_	469.3	479.8	473.8	470.6	-	-
	*T*_DTG_	477.9	488.2	487.9	485.2	-	-
0.5	*T*_5%_	441.8	444.7	449.1	448.7	445.2	445.7
	*T*_onset_	469.3	472.8	462.9	473.2	476.2	470.5
	*T*_DTG_	477.9	487.2	489	487.8	489.8	486.6
1.0	*T*_5%_	441.8	443.7	445.5	445.3	445.3	445.1
	*T*_onset_	469.3	476.2	472.4	460	472.0	471.1
	*T*_DTG_	477.9	487.7	489.6	486.3	490.1	487.2
1.5	*T*_5%_	441.8	-	-	-	445.1	443.7
	*T*_onset_	469.3	-	-	-	476.0	474.4
	*T*_DTG_	477.9	-	-	-	490.3	489.2

**Table 5:** Melting and crystallisation temperatures of obtained composites.

Additive	Concentration of Additive (%)				Concentration of Additive (%)			
	0.1	0.5	1.0	1.5	0.1	0.5	1.0	1.5
	Crystallisation Temperature (°C)				Melting Temperature (°C)			
Neat PE	98.8				112.4			
SSQ-8Cl	99.0	99.5	99.8	-	113.4	112.2	111.8	-
*i*Bu_7_SSQ-Cl	99.3	99.1	99.7	-	112.3	112.5	111.8	-
*i*Bu_7_SSQ-NH_2_	99.9	99.4	100.0	-	112.0	111.8	111.5	-
*i*Bu_7_SSQ-Vi	-	99.5	99.0	98.4	-	112.6	113.1	113.8
*i*Bu_7_SSQ-3OH	-	98.9	99.2	99.8	-	112.7	113.0	112.0

**Table 6:** MFR values for SSQ/PE composites.

Additive	Concentration of Additive (%)			
	0.1	0.5	1.0	1.5
	MFR (g/10 min)			
Neat PE	2.02			
SSQ-8Cl	1.83	1.88	1.90	-
*i*Bu_7_SSQ-Cl	1.87	1.92	1.99	-
*i*Bu_7_SSQ-NH_2_	1.87	1.91	1.98	-
*i*Bu_7_SSQ-Vi	-	1.91	1.99	2.03
*i*Bu_7_SSQ-3OH	-	1.86	1.86	1.89

## Supplementary Materials

The following are available online at https://www.mdpi.com/2073-4360/12/10/2269/s1, Table S1: List of isolated compounds; Table S2. Crystallinity indices (CI) of the obtained composites; Table S3. Heat Deflection Temperatures of the obtained composites; Figure S1: ^1^H NMR of SSQ-8Cl; Figure S2: ^13^C NMR of SSQ-8Cl; Figure S3: ^29^Si NMR of SSQ-8Cl; Figure S4: ^1^H NMR of iBu_7_SSQ-Cl; Figure S5: ^13^C NMR of iBu_7_SSQ-Cl; Figure S6: ^29^Si NMR of iBu_7_SSQ-Cl; Figure S7: ^1^H NMR of iBu_7_SSQ-NH_2_; Figure S8: ^13^C NMR of iBu_7_SSQ-NH_2_; Figure S9: ^29^Si NMR of iBu_7_SSQ-NH_2_; Figure S10: ^1^H NMR of iBu_7_SSQ-Vi; Figure S11: ^13^C NMR of iBu_7_SSQ-Vi; Figure S12: ^29^Si NMR of iBu7SSQ-Vi; Figure S13: ^1^H NMR of iBu_7_SSQ-3OH; Figure S14: ^13^C NMR of iBu_7_SSQ-3OH; Figure S15: ^29^Si NMR of iBu_7_SSQ-3OH; Figure S16: MALDI-TOF-MS spectrogram of iBu_7_SSQ-3OH heat treatment products; Figure S17: MALDI-TOF-MS spectrogram of iBu_7_SSQ-3OH heat treatment products (enhanced); Figure S18: 0.1% SSQ-8Cl/PE; Figure S19: 0.5% SSQ-8Cl/PE; Figure S20: 1% SSQ-8Cl/PE; Figure S21: 0.1% iBu7-SSQ-Cl/PE; Figure S22: 0.5% iBu_7_-SSQ-Cl/PE; Figure S23: 1% iBu_7_-SSQ-Cl/PE; Figure S24: 0.1% iBu_7_-SSQ-NH2/PE; Figure S25: 0.5% iBu_7_-SSQ-NH2/PE; Figure S26: 1% iBu_7_-SSQ-NH2/PE; Figure S27: 0.5% iBu_7_-SSQ-Vi/PE; Figure S28: 1% iBu_7_-SSQ-Vi/PE; Figure S29: 1.5% iBu_7_-SSQ-Vi/PE; Figure S30: 0.5% iBu_7_-SSQ-3OH/PE; Figure S31: 0.5% iBu_7_-SSQ-3OH/PE (higher magnification); Figure S32: 1% iBu_7_-SSQ-3OH/PE; Figure S33: 1% iBu_7_-SSQ-3OH (higher magnification); Figure S34: 1.5% iBu_7_-SSQ-3OH; Figure S35: 1.5% iBu_7_-SSQ-3OH (higher magnification).
