# Supplementary material for: Silsesquioxane Derivatives as Functional Additives for Preparation of Polyethylene-Based Composites: A Case of Trisilanol Melt-Condensation
Source: Polymers (Basel). 2020 Oct 2;12(10):2269. doi: 10.3390/polym12102269 (PMC7601124; doi:10.3390/polym12102269)
Supplement: Supplementary file 1 [file polymers-12-02269-s001.pdf]

*Supplementary Information for*

# Silsesquioxane derivatives as functional additives for preparation of polyethylene-based composites: a case of trisilanol melt-condensation

**Dariusz Brząkalski [1]*, Robert E. Przekop [2], Bogna Sztorch [2], Paulina Jakubowska [3], Marek Jałbrzykowski [4] and Bogdan Marciniec [1,2]**

[1] Faculty of Chemistry, Adam Mickiewicz University in Poznań, Uniwersytetu Poznańskiego 8, 61-614 Poznań, Poland
[2] Centre for Advanced Technologies, Adam Mickiewicz University in Poznań, Uniwersytetu Poznańskiego 10, 61-614 Poznań, Poland
[3] Poznan University of Technology, Faculty of Chemical Technology, Berdychowo 4, 60-695 Poznań, Poland
[4] Bialystok University of Technology, Faculty of Mechanical Engineering, Wiejska 45 C, 15-351 Bialystok, Poland
*Corresponding Author: D.B.: e-mail: dariusz.brzakalski@amu.edu.pl

## Table of contents:

1. List of isolated compounds ........................................ - 2 -

2. Characterization data of the products: ........................................ 3

3. MALDI-TOF-MS analysis of $iBu_7SSQ$-3OH heat treatment products ........................................ 13

4. SEM and EDS images of the SSQ/PE composites ........................................ 14

5. Data Tables ........................................ 32

## 1. List of isolated compounds

**Table S1: List of isolated compounds:**

| **Structure** | **Compound #** | **Code:** |
|---|---|---|
| 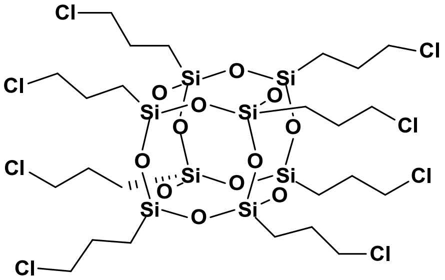 | **1** | SSQ-8Cl |
| 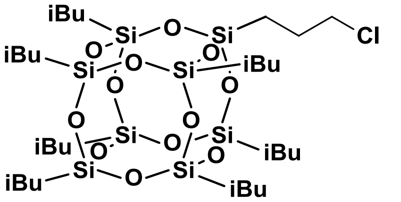 | **2** | iBu$_7$SSQ-Cl |
| 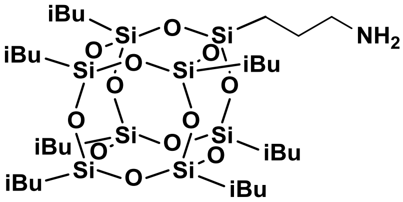 | **3** | iBu$_7$SSQ-$NH_2$ |
| 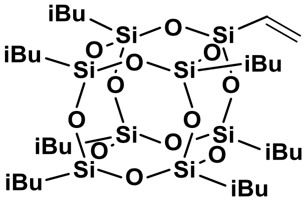 | **4** | iBu$_7$SSQ-Vi |
| 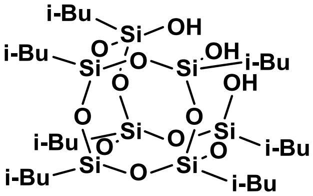 | **5** | iBu$_7$SSQ-3OH |

## 2. Characterization data of the products:

**1,3,5,7,9,11,13,15-octakis(3-chloropropyl)-pentacyclo[9.5.1.1$^{3,9}$.1$^{5,15}$.1$^{7,13}$]octasiloxane (2)**

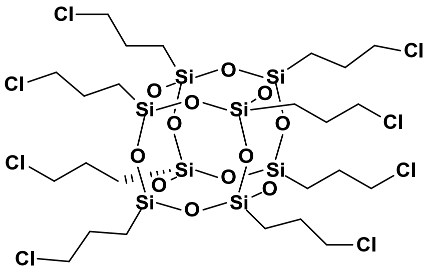

$^{1}$**H NMR** (400 MHz, $CDCl_3$): δ (ppm) = 3.54 (t, J = 6.7Hz, 16H, Si-$CH_2CH_2\underline{CH_2}$-Cl), 1.88 (p, J = 6.7Hz, 16H, Si-$CH_2\underline{CH_2}CH_2$-Cl), 0.80 (t, J = 8.2Hz, 16H, Si-$\underline{CH_2}CH_2CH_2$-Cl);
$^{13}$**C NMR** (101 MHz, $CDCl_3$): δ (ppm) = 47.17 (Si-$CH_2CH_2\underline{CH_2}$-Cl), 26.39 (Si-$CH_2\underline{CH_2}CH_2$-Cl), 9.48 (Si-$\underline{CH_2}CH_2CH_2$-Cl);
$^{29}$**Si NMR** (79,5 MHz, $CDCl_3$): δ (ppm) = -67.07.
**FT-IR (ATR)** = 2994, 2953-2874, 1456, 1436, 1408, 1349, 1311, 1272, 1240, 1190, 1081, 1000, 914, 867, 811, 778-649, 551, 534.

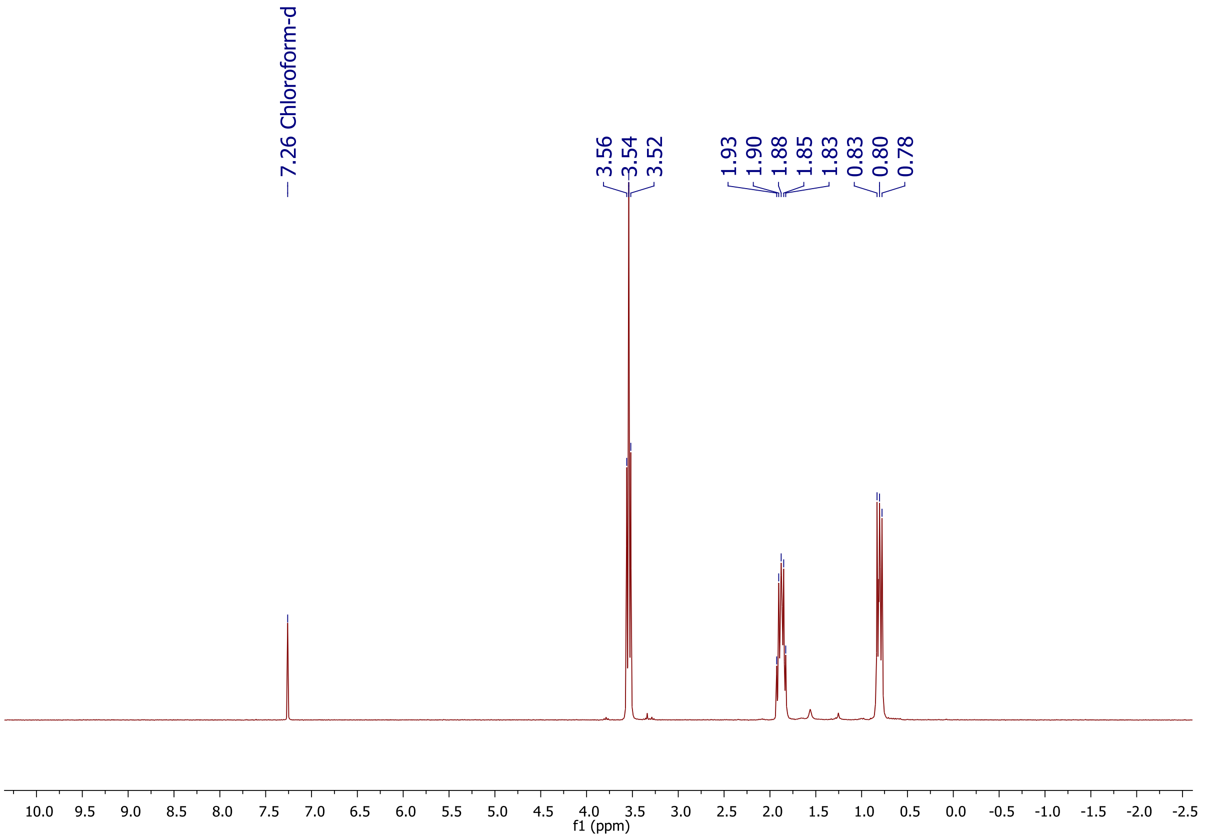


Figure S1: $^{1}$H NMR of SSQ-8Cl

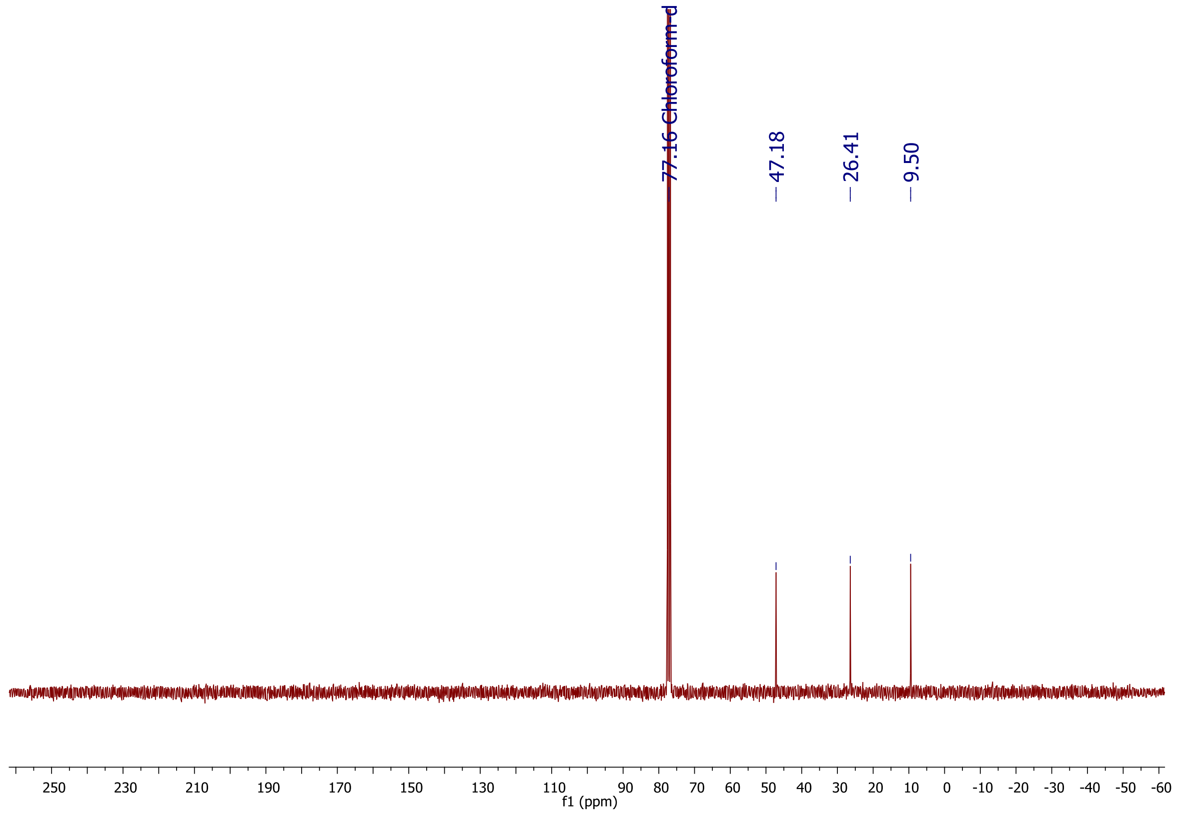


Figure S2: $^{13}$C NMR of SSQ-8Cl

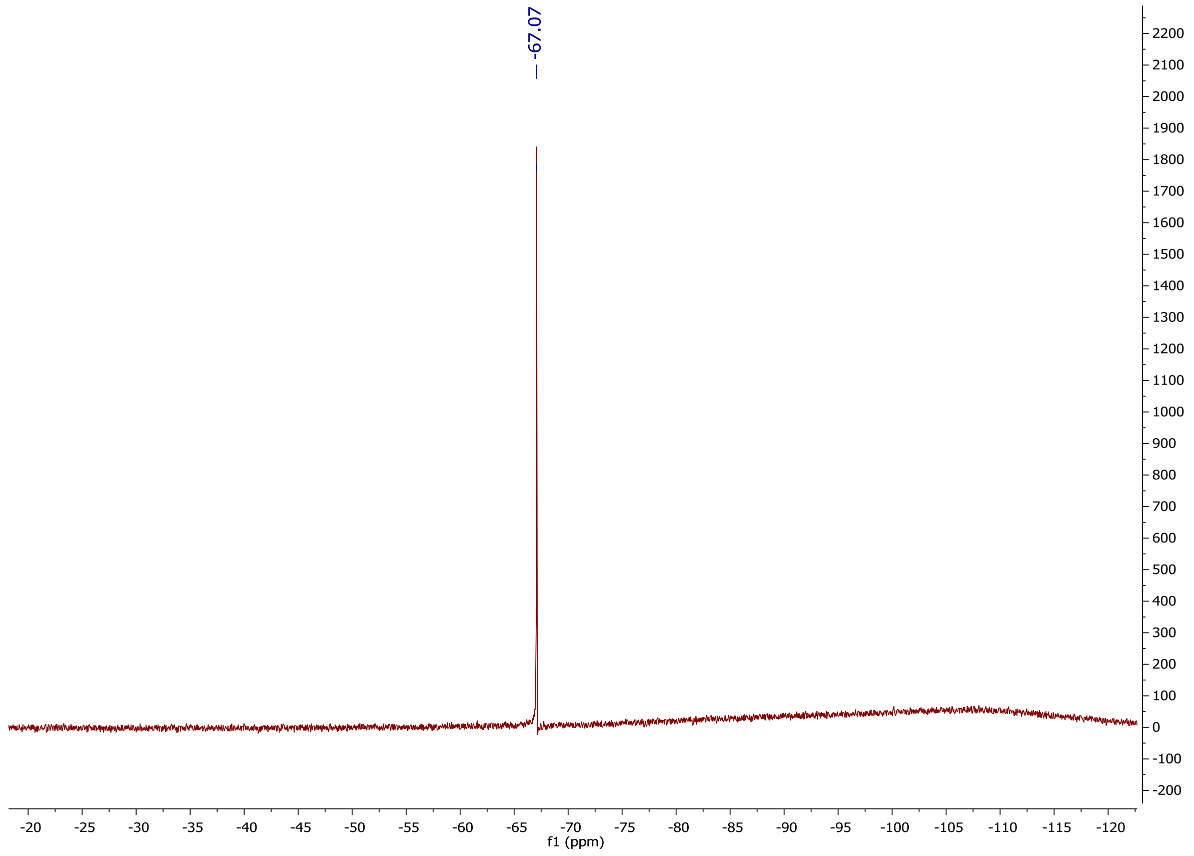


Figure S3: $^{29}$Si NMR of SSQ-8Cl

**1-(3-chloropropyl)-3,5,7,9,11,13,15-heptaisobutylpentacyclo[9.5.1.1$^{3,9}$.1$^{5,15}$.1$^{7,13}$]octasiloxane (2)**

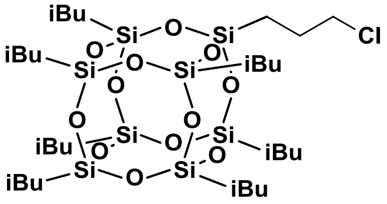


$^{1}$**H NMR** (400 MHz, $CDCl_3$): δ (ppm) = 3.52 (t, J = 8.1Hz, 2H, $-CH_2CH_2C\underline{H_2}Cl$), 1.92-1.79 (m, 9H, $-CH_2C\underline{H}(CH_3)_2$, $-CH_2C\underline{H_2}CH_2Cl$), 0.96 (d, J = 6.6Hz, 42H, $-CH_2CH(C\underline{H_3})_2$), 0.74 (t, J = 8.1Hz, 2H, $-C\underline{H_2}CH_2CH_2Cl$), 0.62-0.59 (m, 14H, $-C\underline{H_2}CH(CH_3)_2$);
$^{13}$**C NMR** (101 MHz, $CDCl_3$): δ (ppm) = 47.41 ($-CH_2CH_2\underline{CH_2}Cl$), 26.63 $-CH_2\underline{CH_2}CH_2Cl$), 25.85, 24.03, 22.65, 22.59 (iBu), 9.94 ($-C\underline{H_2}CH_2CH_2Cl$);
$^{29}$**Si NMR** (79,5 MHz, $CDCl_3$): δ (ppm) = -67.59, -67.87, -68.12.
**FT-IR (ATR)** = 2953, 2928, 2905, 2871, 1464, 1398, 1383, 1366, 1332, 1229, 1168, 1081, 955, 916, 838, 804, 745, 680, 556.

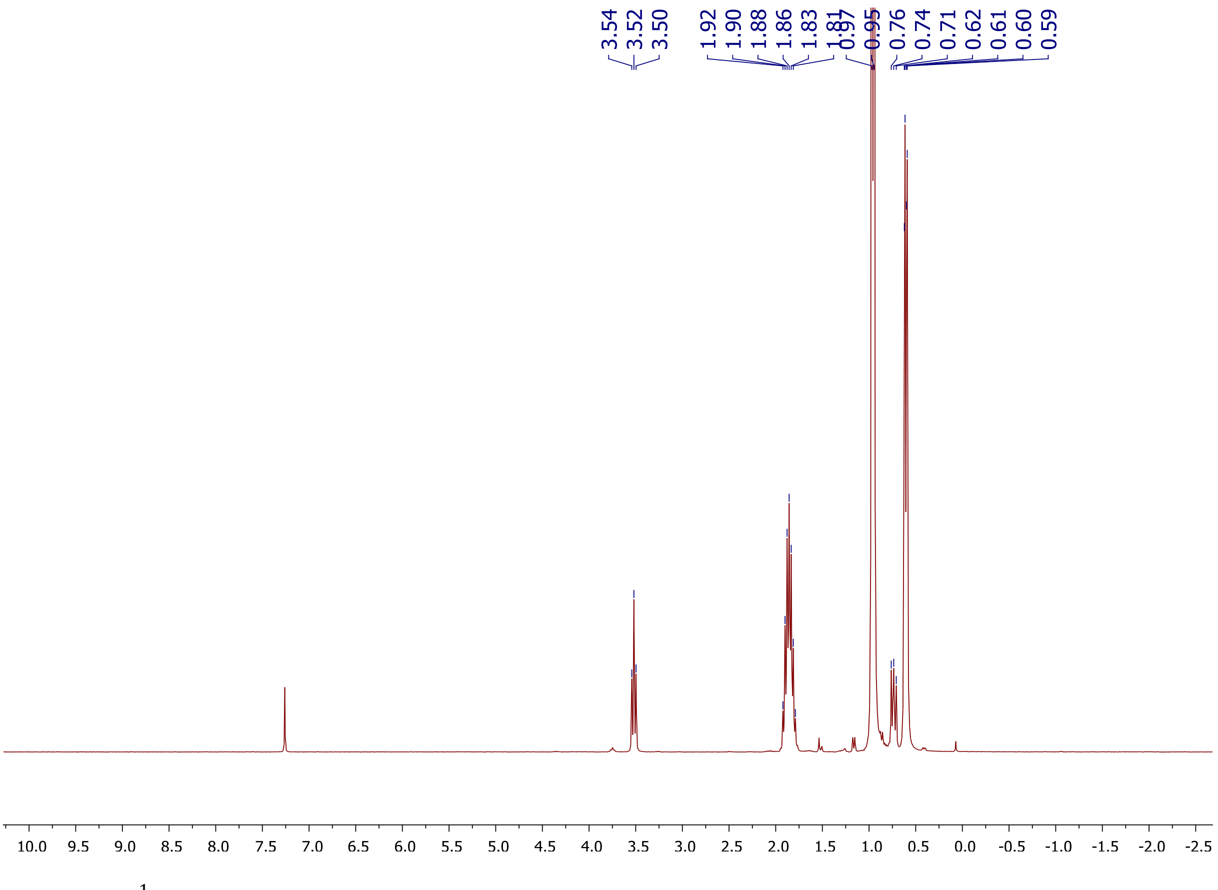


Figure S4: $^{1}$H NMR of $iBu_7SSQ$-Cl

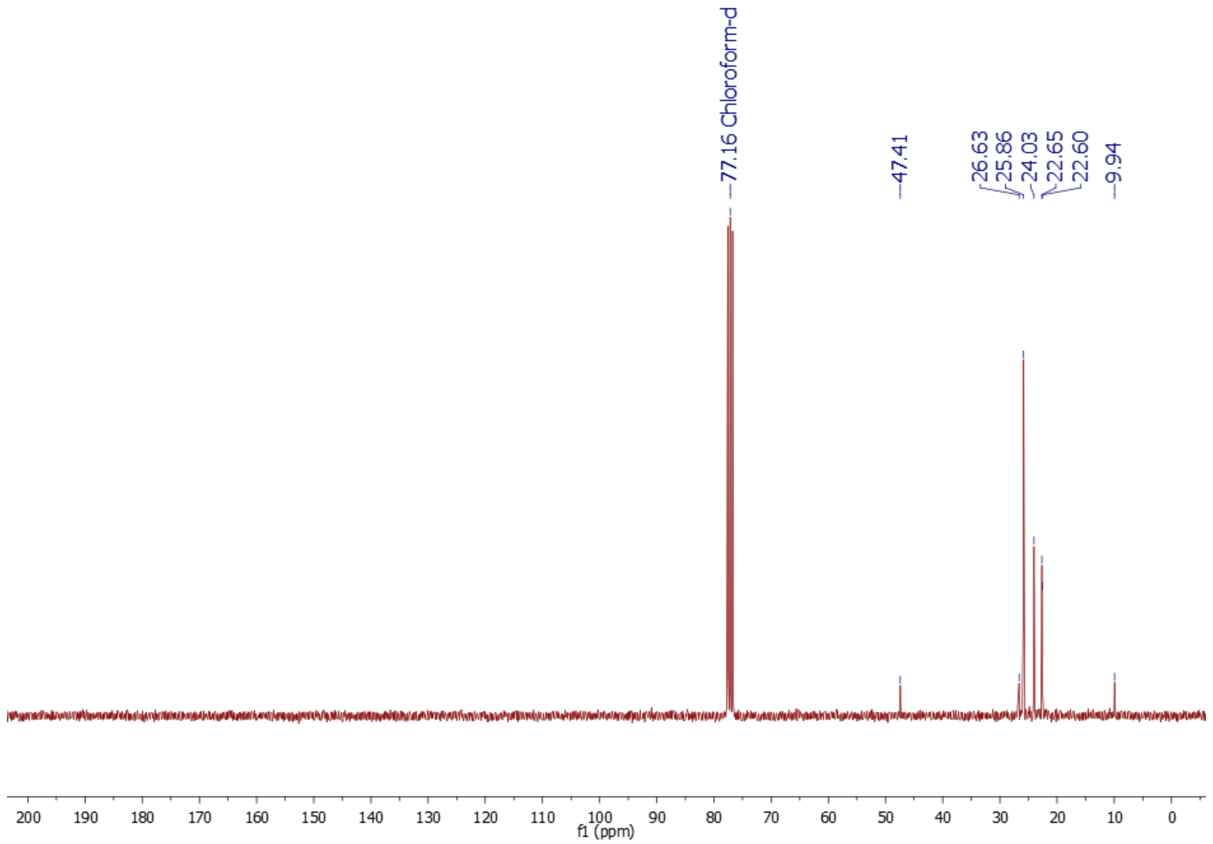


Figure S5: $^{13}C$ NMR of $iBu_7SSQ$-Cl

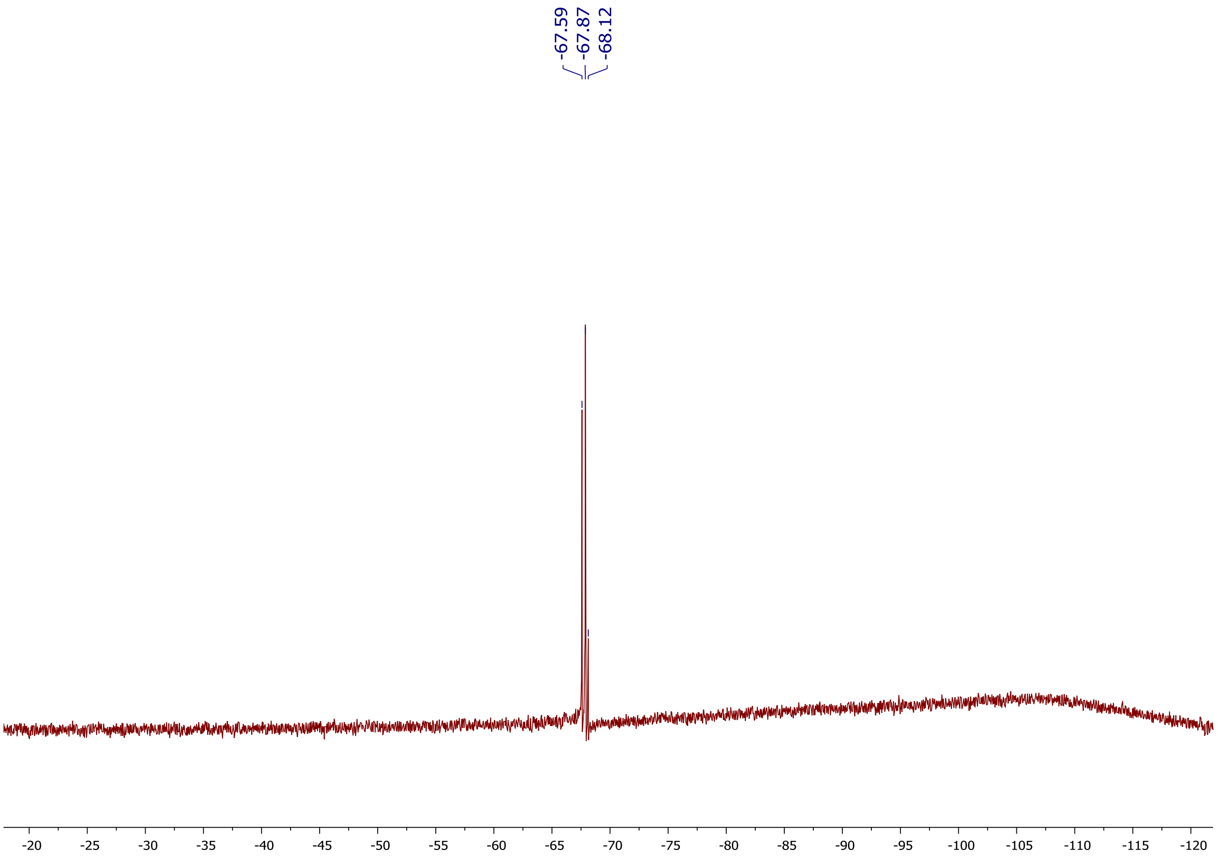


Figure S6: $^{29}Si$ NMR of $iBu_7SSQ$-Cl

**1-(3-aminopropyl)-3,5,7,9,11,13,15-heptaisobutylpentacyclo[9.5.1.1$^{3,9}$.1$^{5,15}$.1$^{7,13}$]octasiloxane (3)**

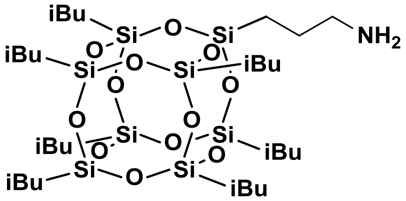


$^{1}$**H NMR** (400 MHz, $CDCl_3$): δ (ppm) = 2.67 (t, J = 7.1Hz, 2H, $-CH_2CH_2C\underline{H_2}NH_2$), 1.90-1.80 (m, 7H, $-CH_2C\underline{H}(CH_3)_2$), 1.53 (p, J = 7.1Hz, 2H, $-CH_2C\underline{H_2}CH_2NH_2$), 0.95 (d, J = 6.6Hz, 42H, $-CH_2CH(C\underline{H_3})_2$), 0.61-0.59 (m, 16H, $-C\underline{H_2}CH(CH_3)_2$, $-C\underline{H_2}CH_2CH_2NH_2$);
$^{13}$**C NMR** (101 MHz, $CDCl_3$): δ (ppm) = 44.96 ($-CH_2CH_2\underline{CH_2}NH_2$), 27.34 ($-CH_2\underline{CH_2}CH_2NH_2$), 25.85, 25.83, 24.04, 24.01, 22.66, 26.63 (iBu), 9.36 ($-\underline{CH_2}CH_2CH_2NH_2$);
$^{29}$**Si NMR** (79,5 MHz, $CDCl_3$): δ (ppm) =.-67.25, -67.70, -67.89;
**IR (ATR)** = 2953, 2928, 2906, 2871, 1464, 1398, 1383, 1366, 1332, 1228, 1168, 1085, 955, 838, 804, 746, 683, 556.

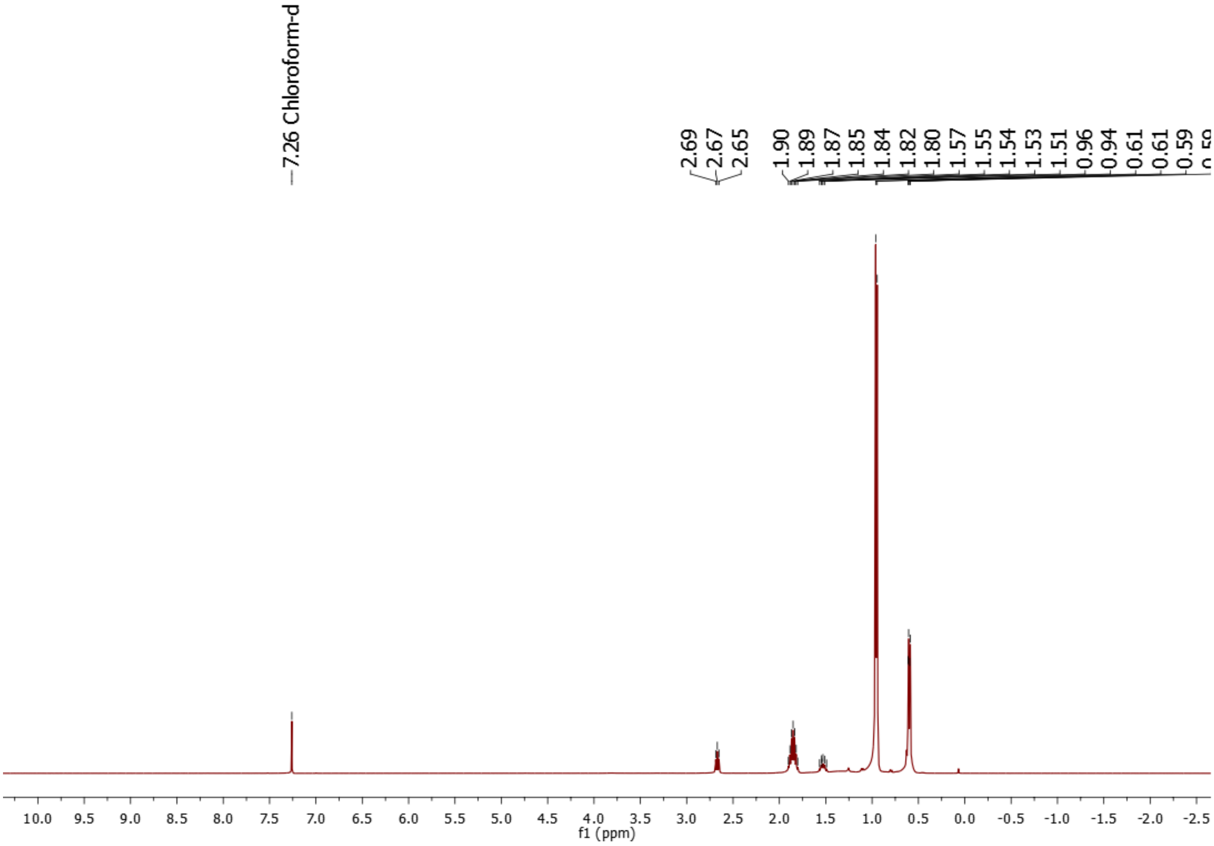


Figure S7: $^{1}$H NMR of $iBu_7SSQ-NH_2$

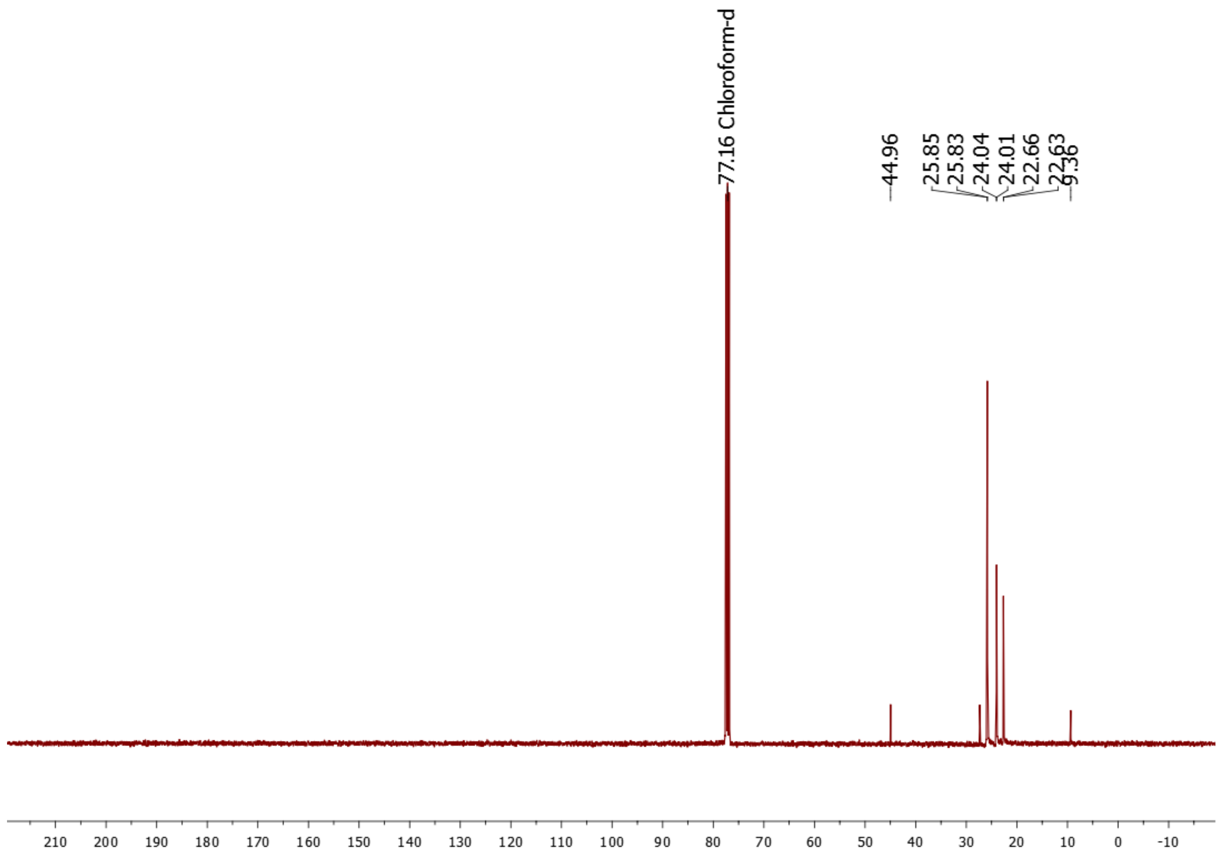


Figure S8: $^{13}C$ NMR of $iBu_7SSQ$-$NH_2$

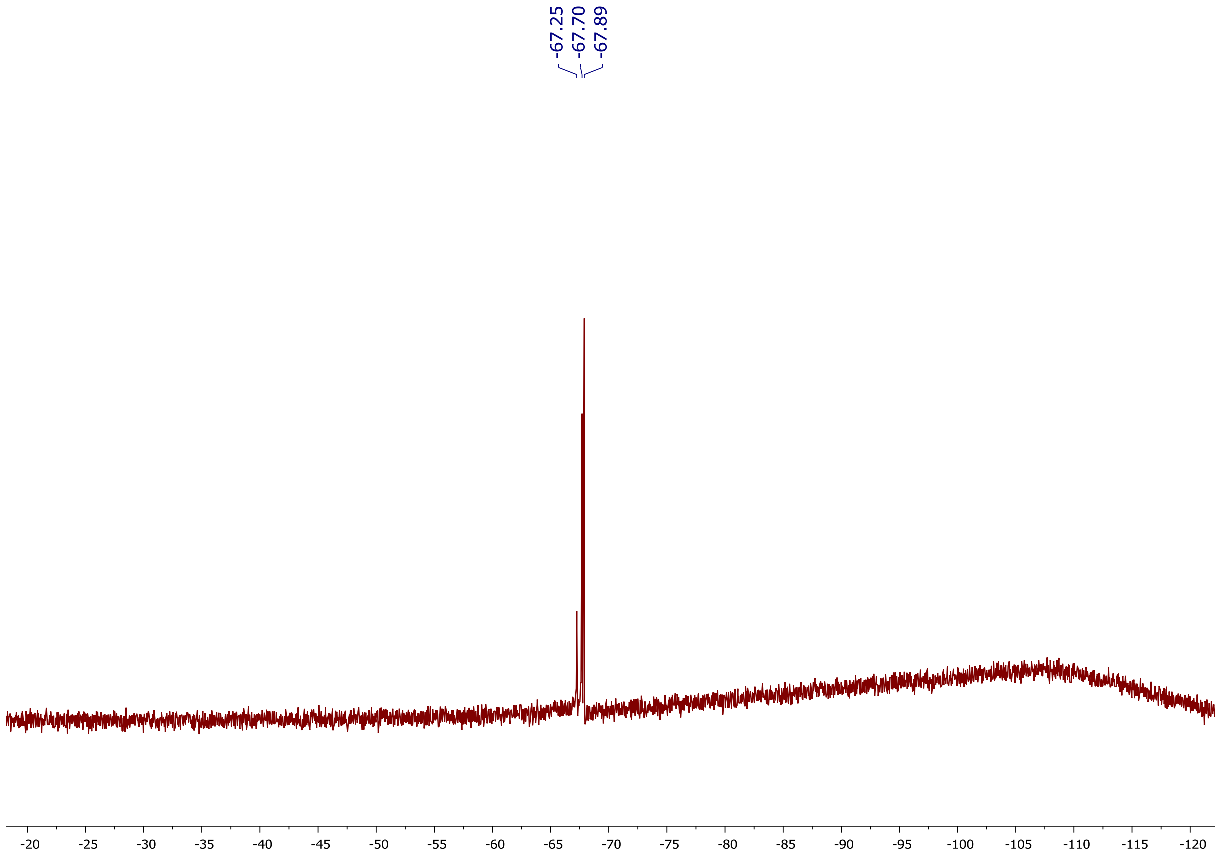


Figure S9: $^{29}Si$ NMR of $iBu_7SSQ$-$NH_2$

**1-vinyl-3,5,7,9,11,13,15-heptaisobutylpentacyclo[9.5.1.1$^{3,9}$.1$^{5,15}$.1$^{7,13}$]octasiloxane (4)**

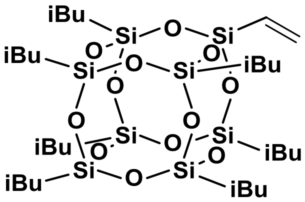


$^{1}$**H NMR** (400 MHz, $CDCl_3$): δ (ppm) = 6.08-5.81 (m, 3H, Vi), 1.93-1.79 (m, 7H, -$CH_2C\underline{H}(CH_3)_2$), 0.96 (d, J = 6.6Hz, 42H, -$CH_2CH(C\underline{H_3})_2$), 0.63-0.59 (m, 14H, -$C\underline{H_2}CH(CH_3)_2$);
$^{13}$**C NMR** (101 MHz, $CDCl_3$): δ (ppm) = 136.00, 130.06 (Vi), 25.87, 25.83, 24.01, 22.64, 22.54 (iBu);
$^{29}$**Si NMR** (79,5 MHz, $CDCl_3$): δ (ppm) = -67.40, -67.87, -81.54.
**IR (ATR)** = 2954, 2928, 2907, 2871, 1464, 1401, 1383, 1366, 1332, 1229, 1168, 1084, 964, 838, 802, 739, 706, 685, 557.

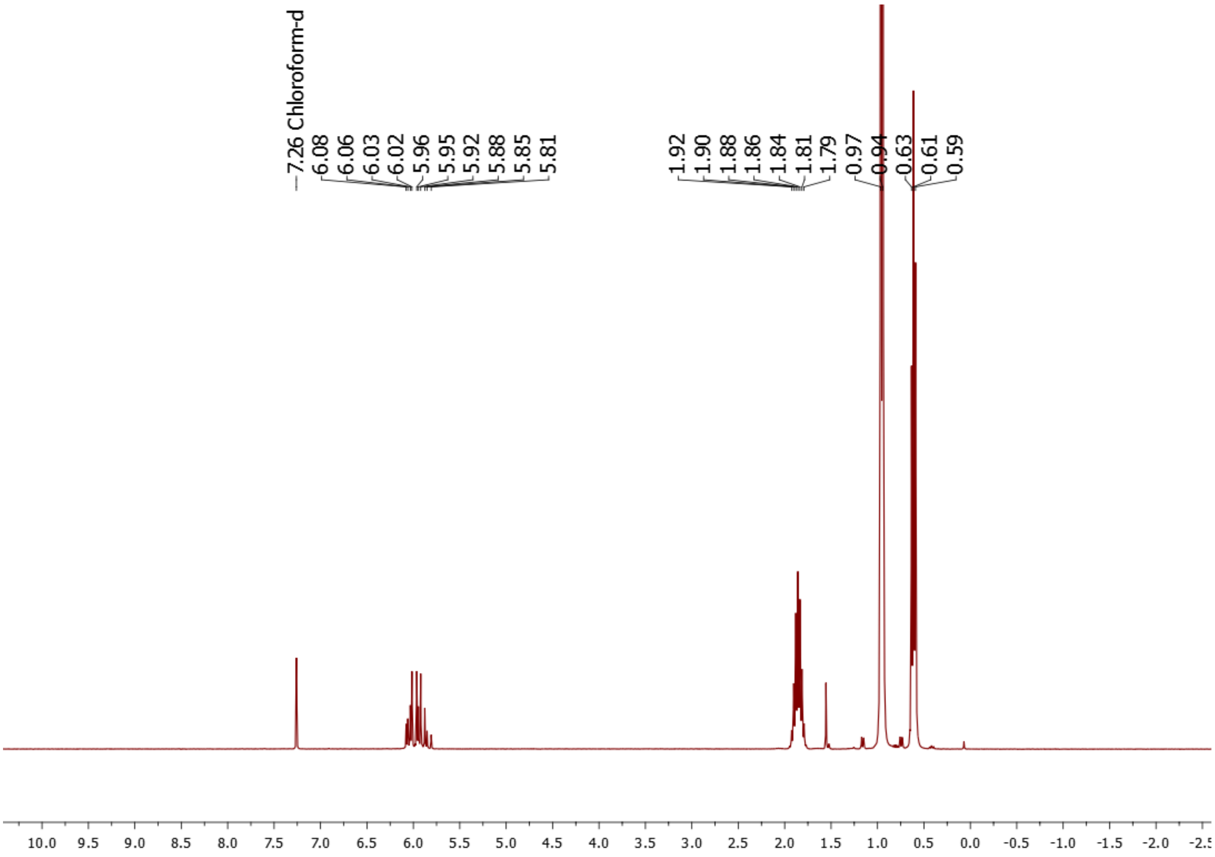


Figure S10: $^{1}$H NMR of $iBu_7SSQ$-Vi

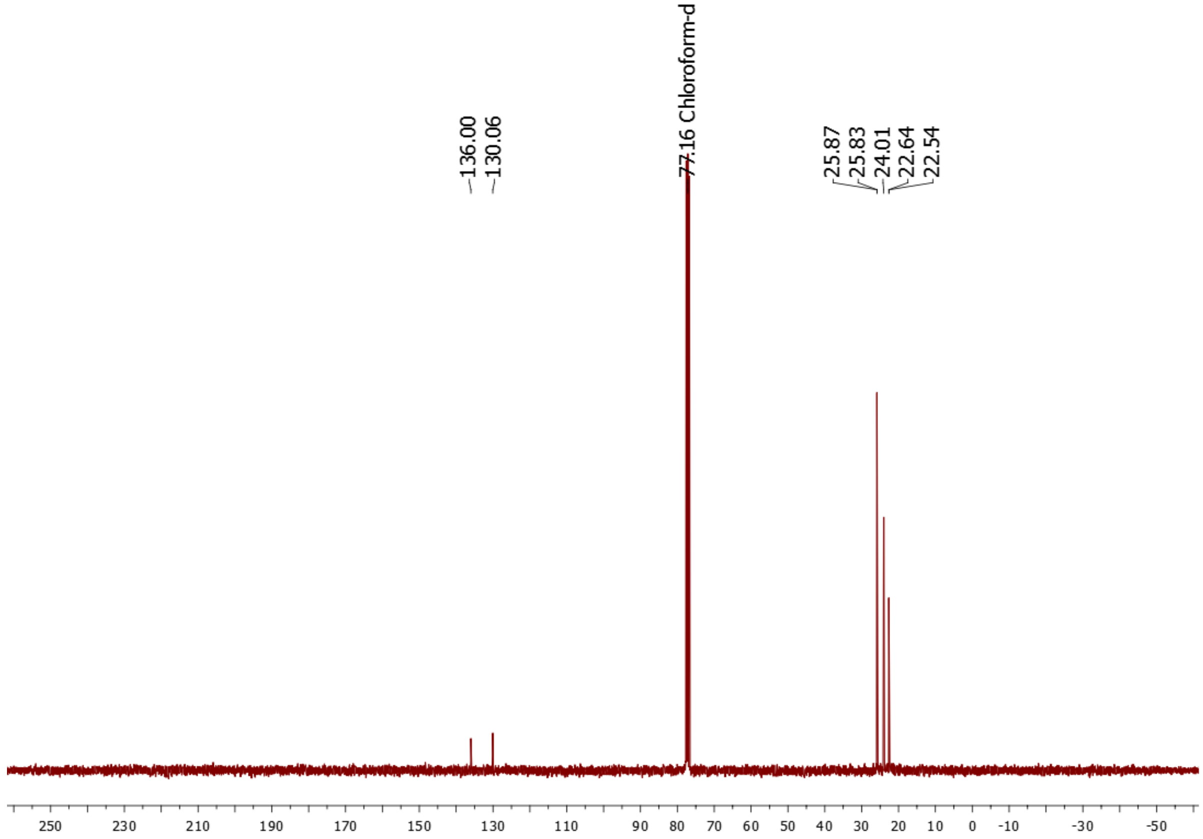


Figure S11: $^{13}$C NMR of $iBu_7SSQ$-Vi

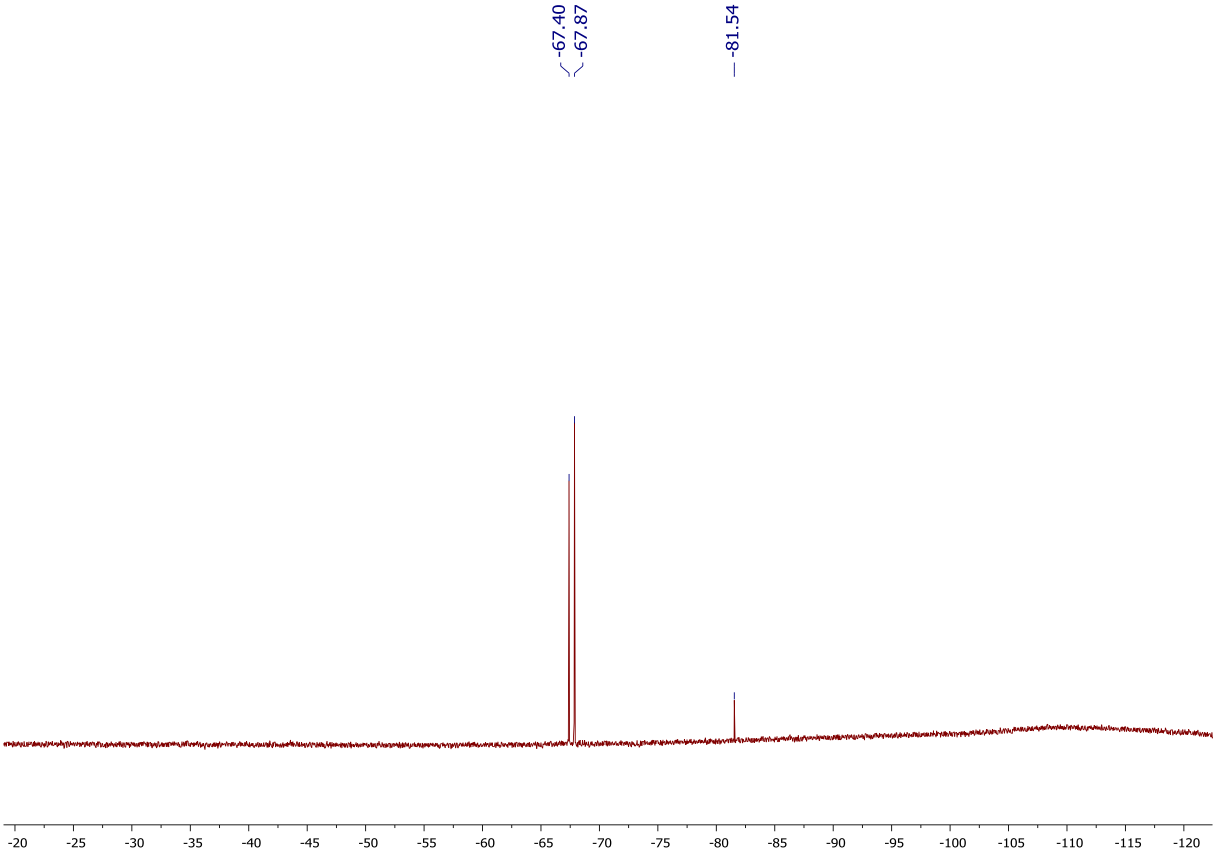


Figure S12: $^{29}$Si NMR of $iBu_7SSQ$-Vi

**1,3,5,7,9,11,13-heptaisobutyltricyclo[5.5.1.1$^{3,9}$.1$^{7,13}$]heptasiloxane-1,5,13-triol (3)**

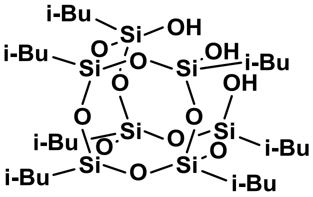


**$^{1}$H NMR** (400 MHz, $CDCl_3$): δ (ppm) = 5.71 (s, 3H, -OH), 1.92-1.77 (m, 7H, (-$CH_2C\underline{H}(CH_3)_2$), 0.97-0.94 (m, 42H, -$CH_2CH(C\underline{H_3})_2$), 0.61-0.56 (m, 14H, -$C\underline{H_2}CH(CH_3)_2$);
**$^{13}$C NMR** (101 MHz, $CDCl_3$): δ (ppm) = 25.94, 25.90, 25.80, 24.10, 24.07, 23.36, 22.98, 22.63;
**$^{29}$Si NMR** (79,5 MHz, $CDCl_3$): δ (ppm) = -58.90, -67.41, -68.71.
**IR (ATR)** = 2953, 2928, 2906, 2871, 1464, 1398, 1383, 1366, 1332, 1228, 1168, 1085, 955, 838, 804, 746, 683, 556.

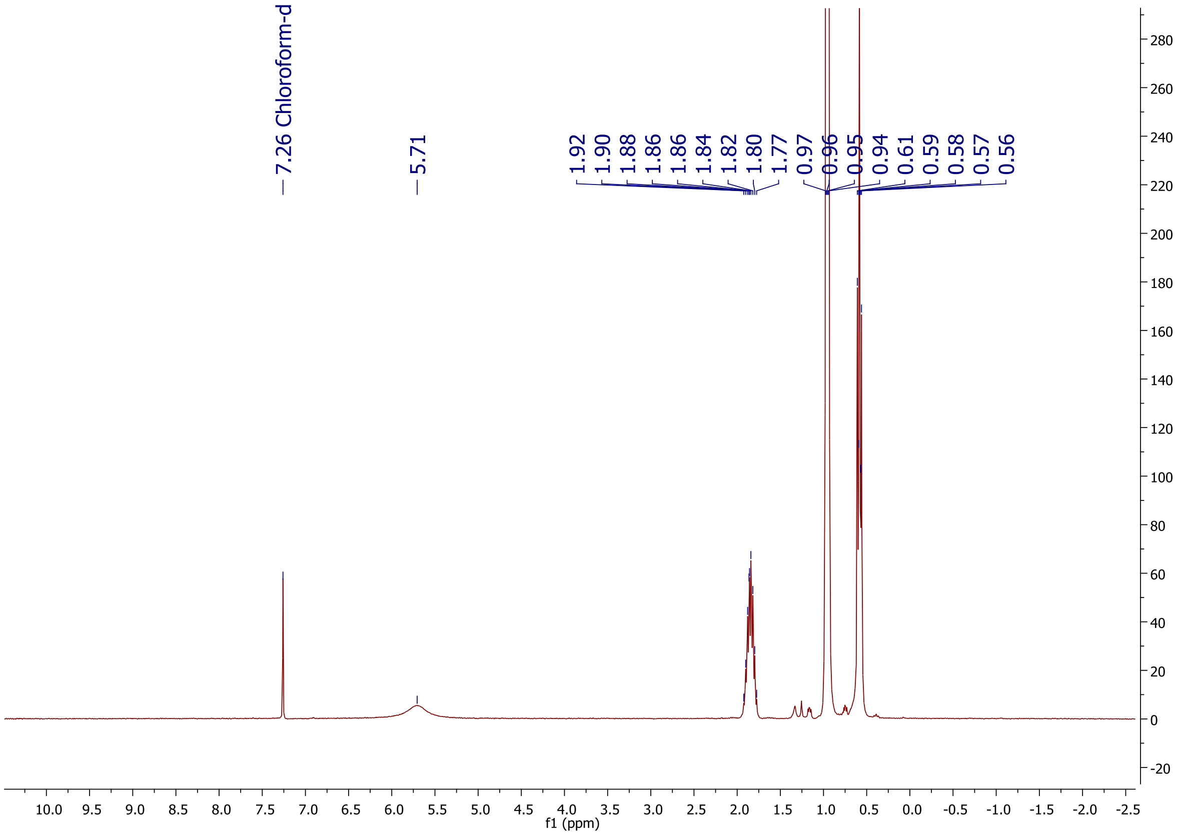


Figure S13: $^{1}$H NMR of $iBu_7SSQ$-3OH

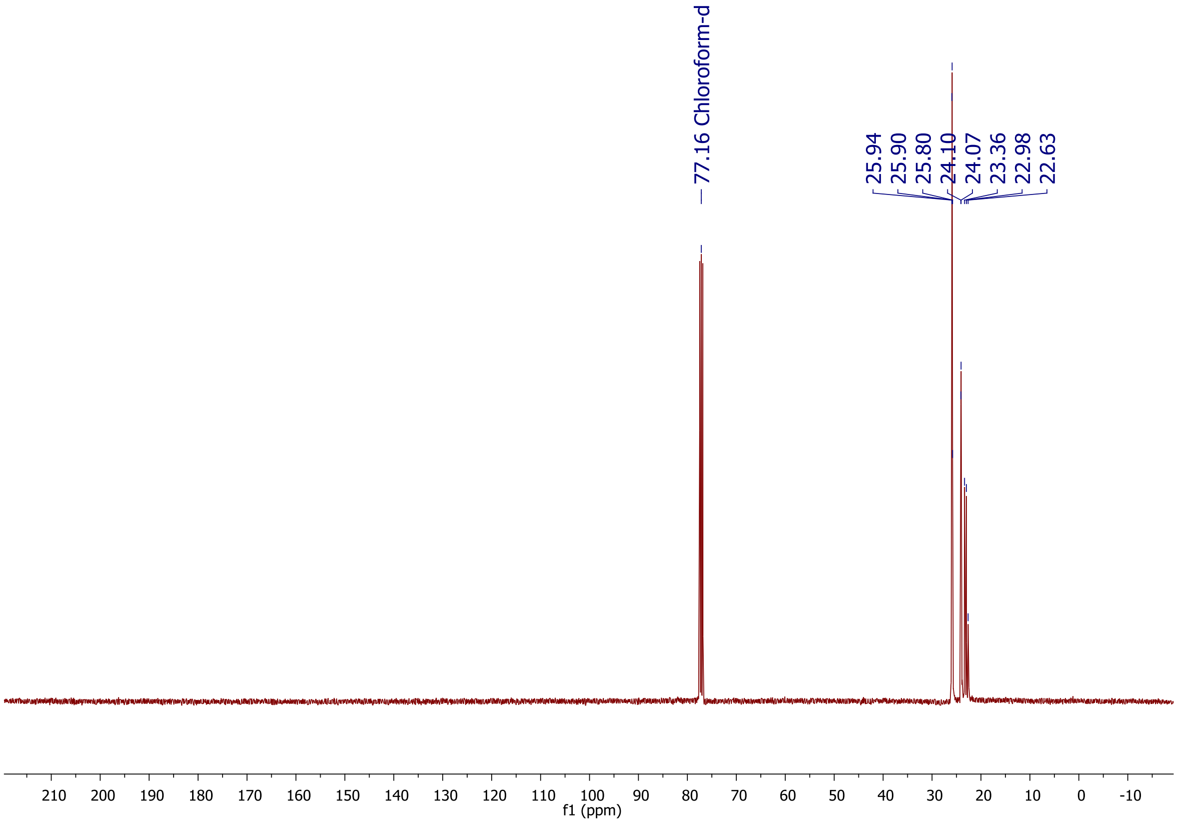


Figure S14: $^{13}$C NMR of iBu$_7$SSQ-3OH

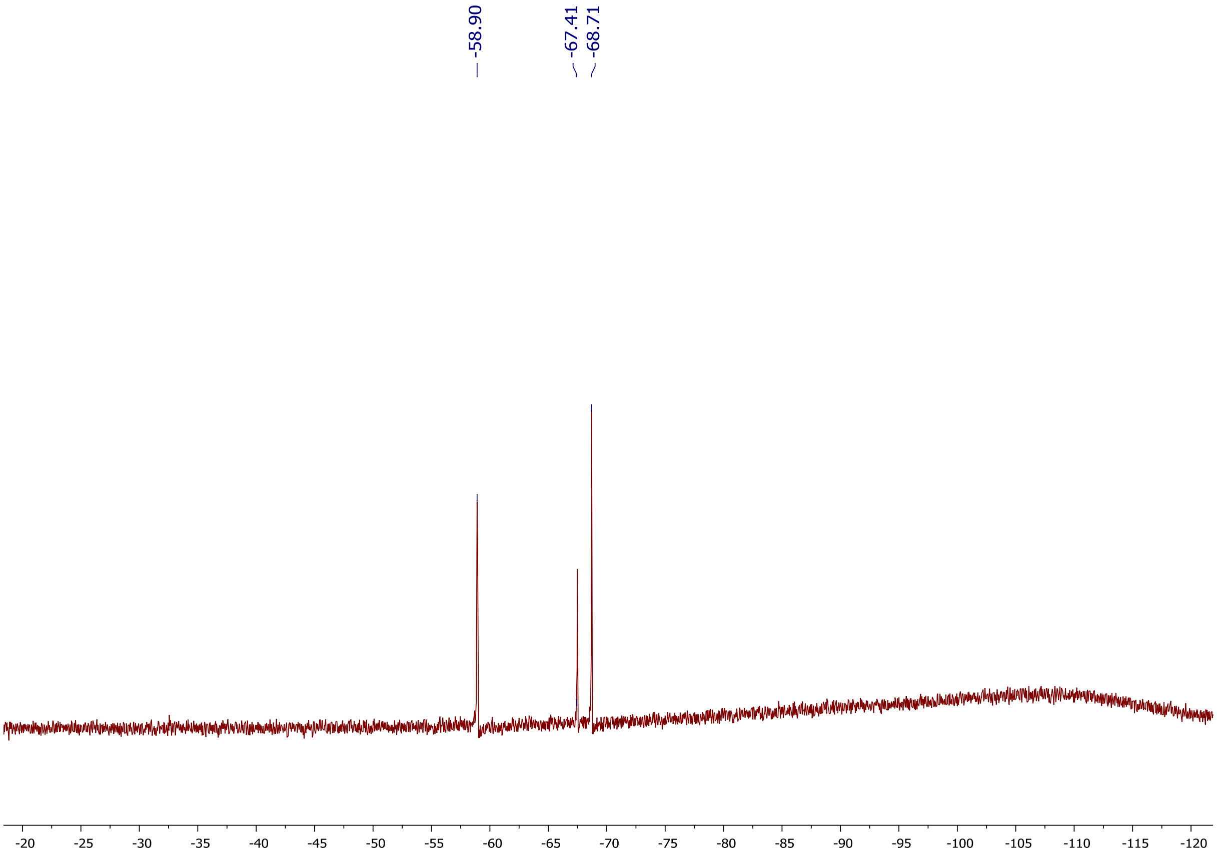


Figure S15: $^{29}$Si NMR of iBu$_7$SSQ-3OH

## 3. MALDI-TOF-MS analysis of $iBu_7SSQ$-3OH heat treatment products

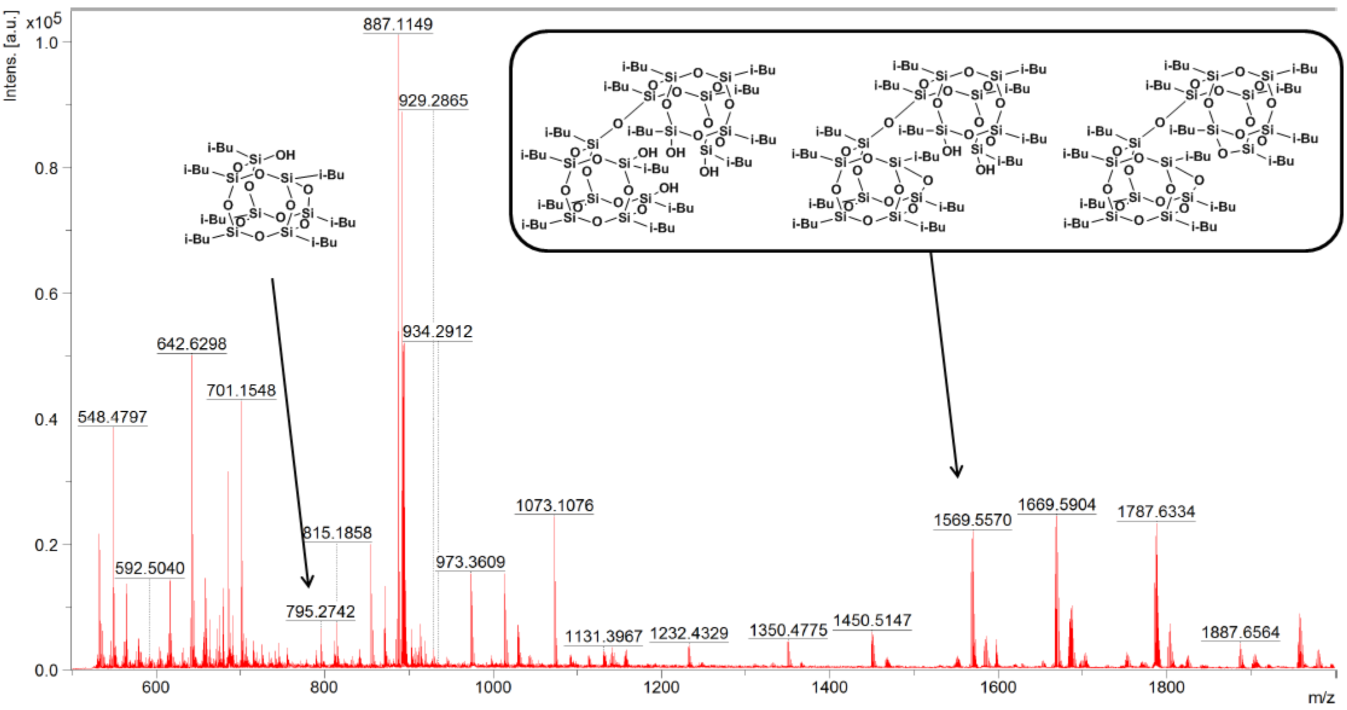

Figure S16: MALDI-TOF-MS spectrogram of $iBu_7SSQ$-3OH heat treatment products

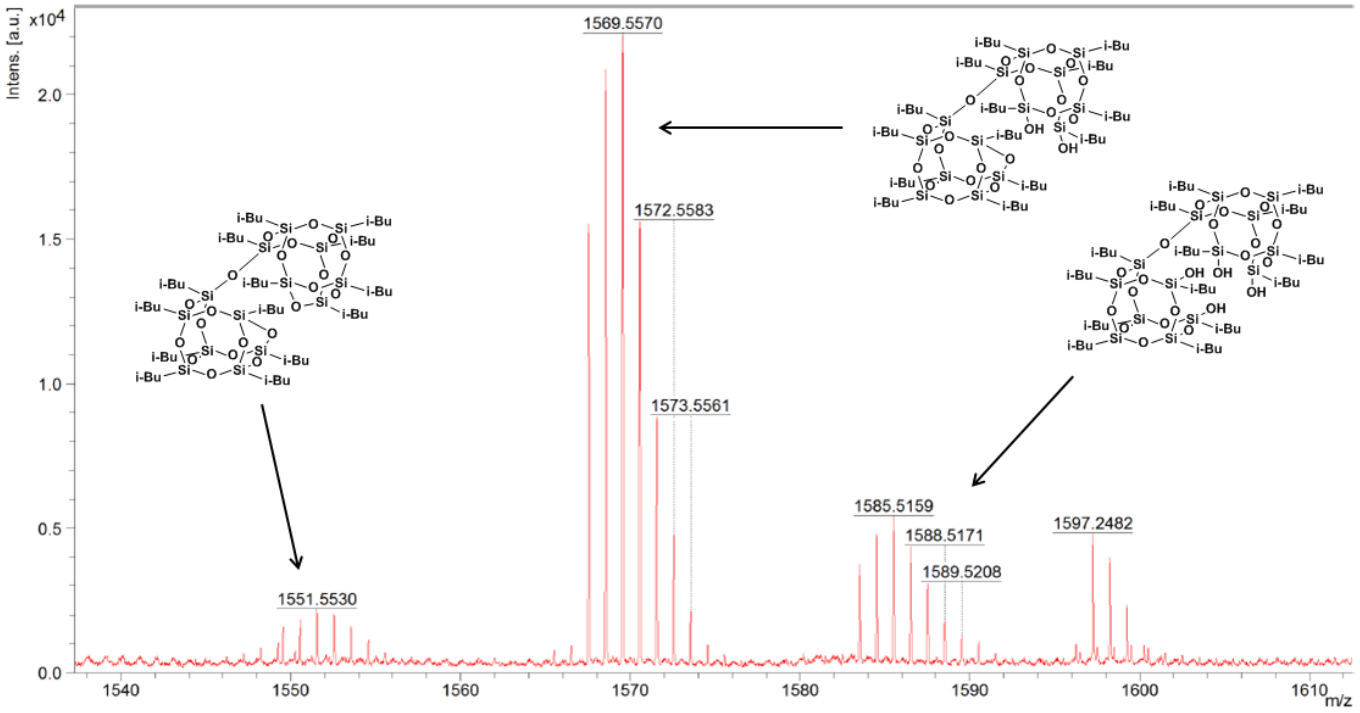

Figure S17: MALDI-TOF-MS spectrogram of $iBu_7SSQ$-3OH heat treatment products (enhanced)

## 4. SEM and EDS images of the SSQ/PE composites

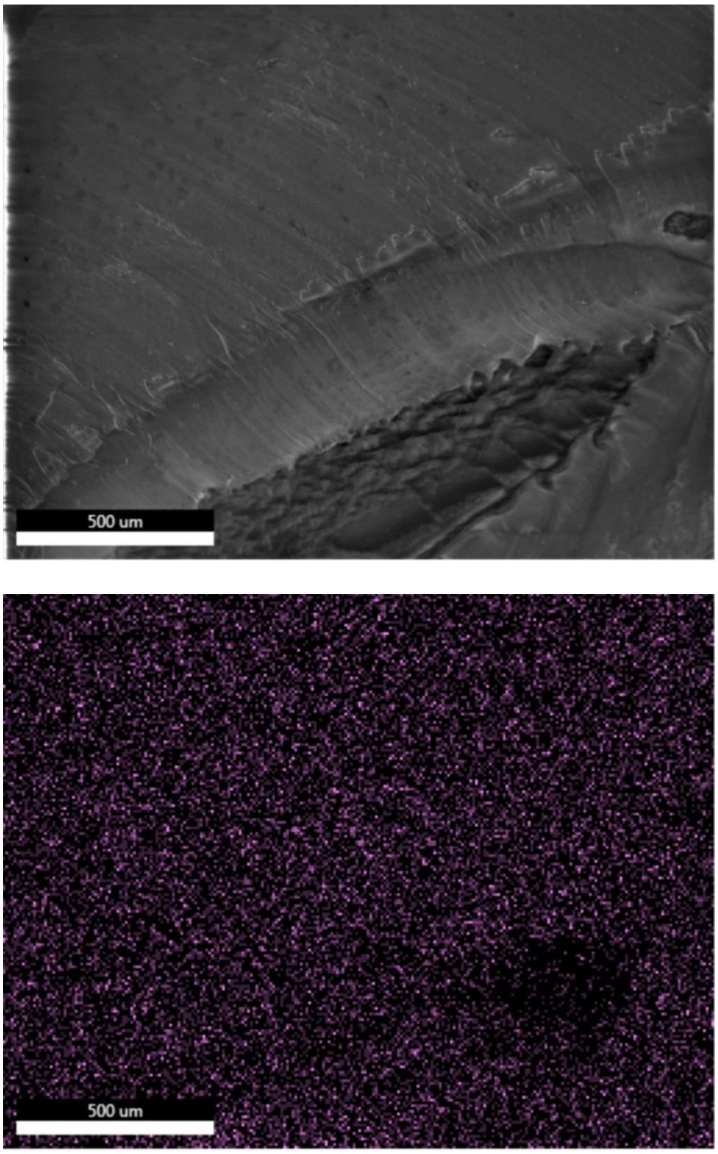


Figure S18: 0.1% SSQ-8Cl/PE

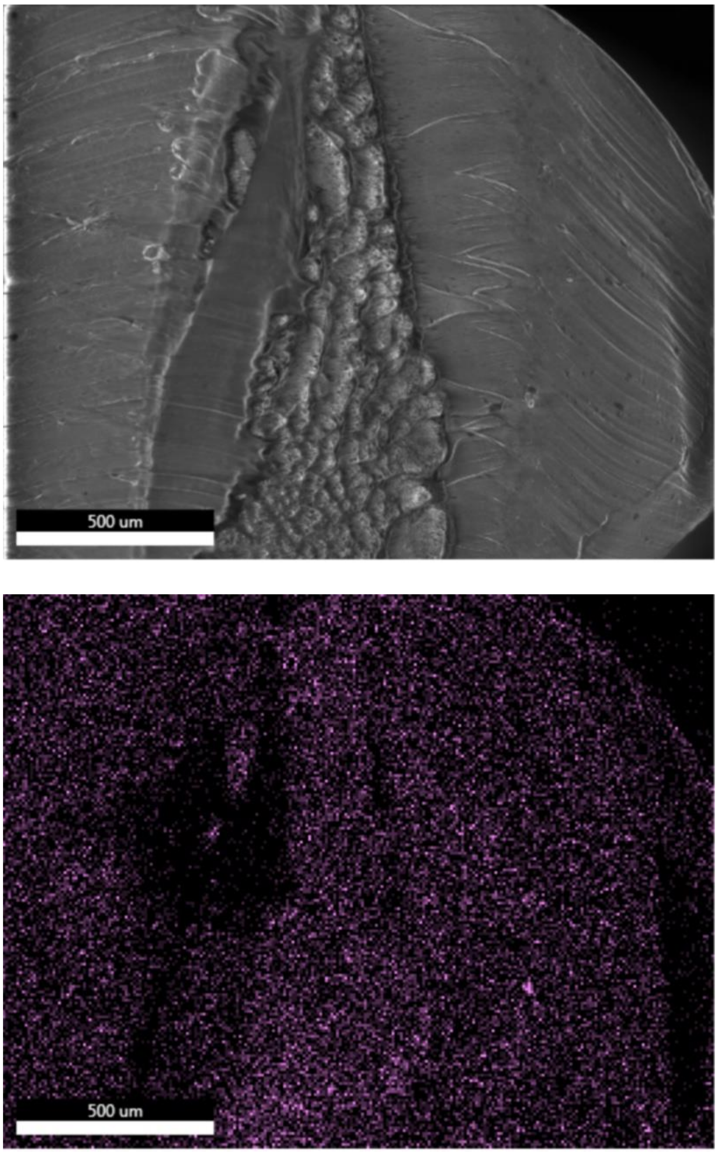


Figure S19: 0.5% SSQ-8Cl/PE

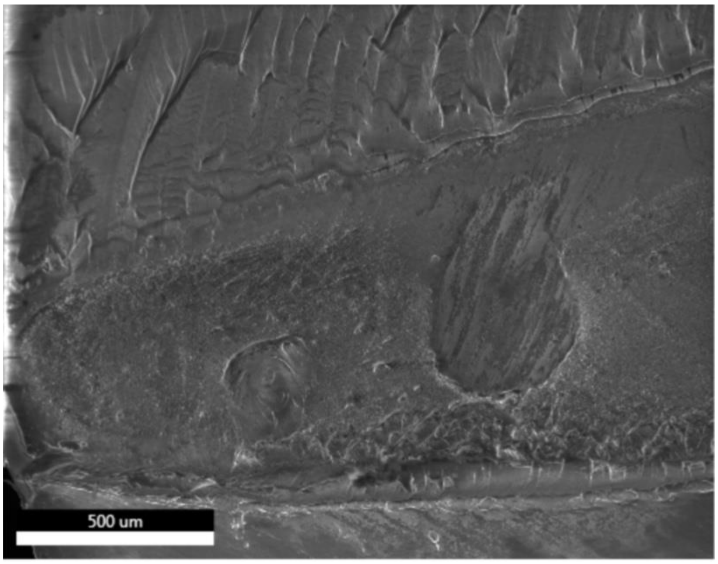


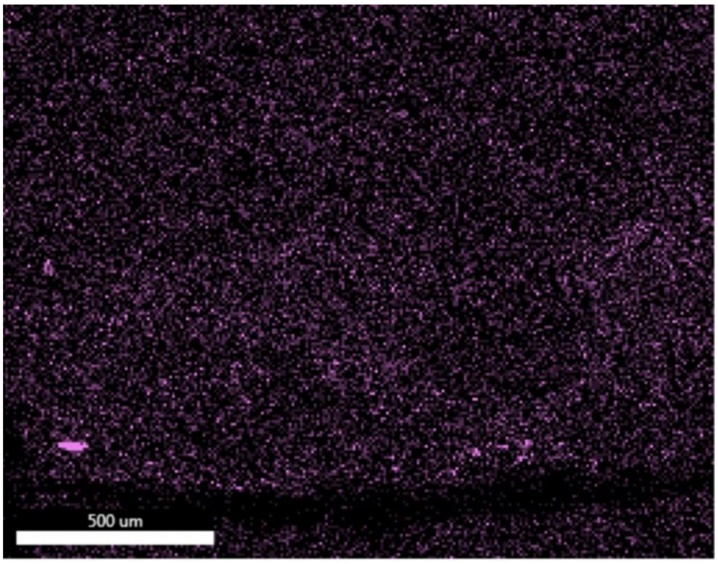


Figure S20: 1% SSQ-8Cl/PE

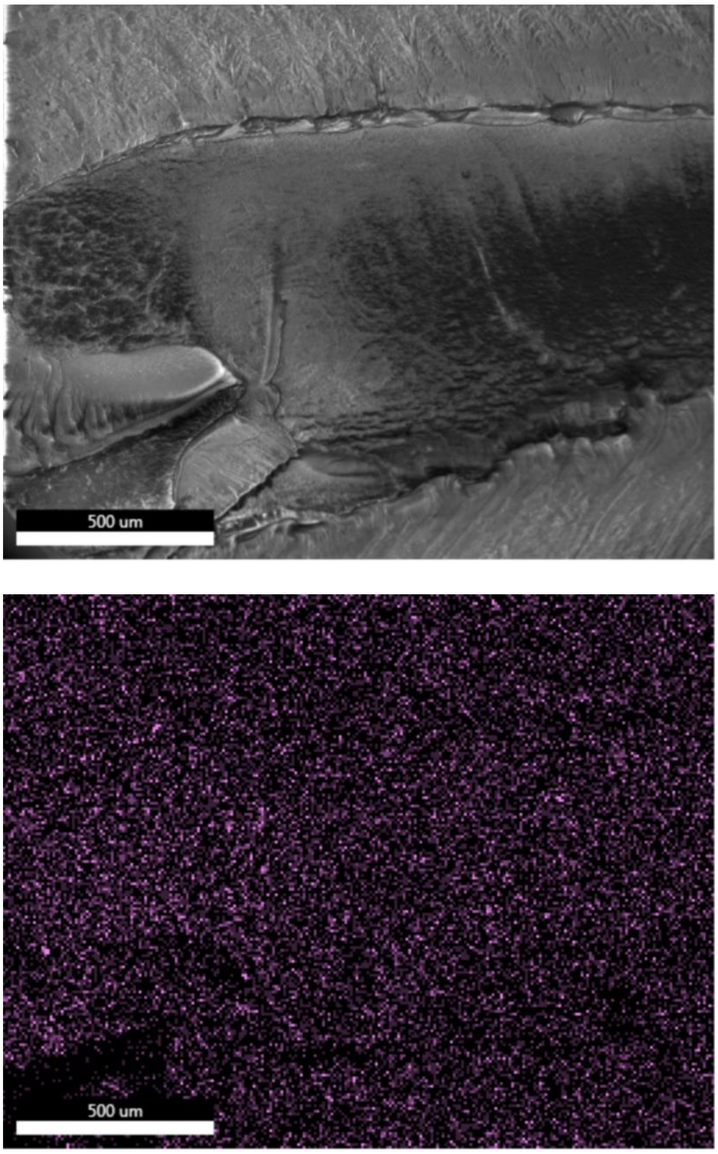


Figure S21: 0.1% $iBu_7$-SSQ-Cl/PE

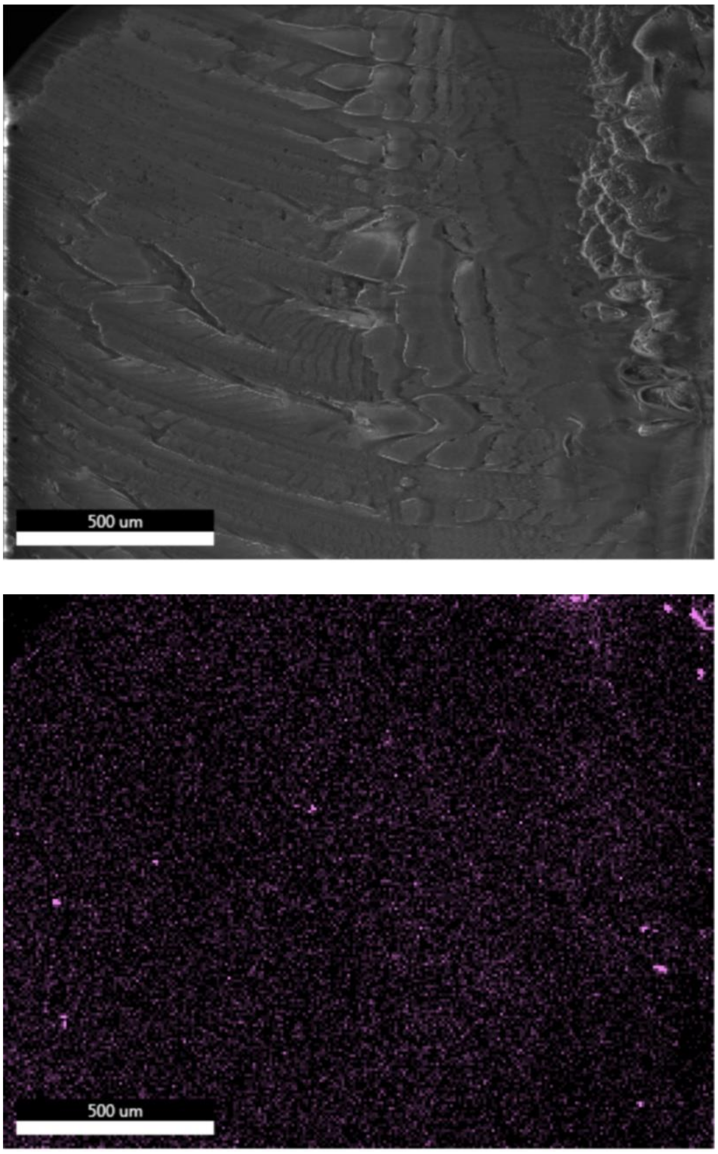


Figure S22: 0.5% $iBu_7$-SSQ-Cl/PE

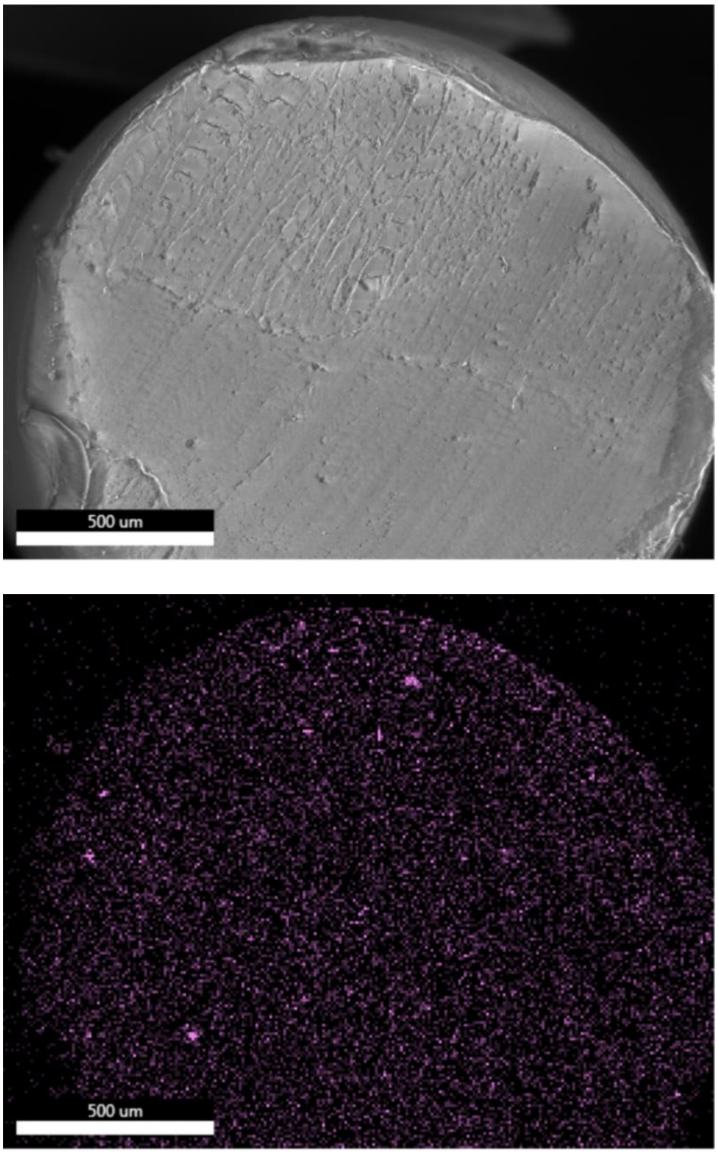


Figure S23: 1% $iBu_7$-SSQ-Cl/PE

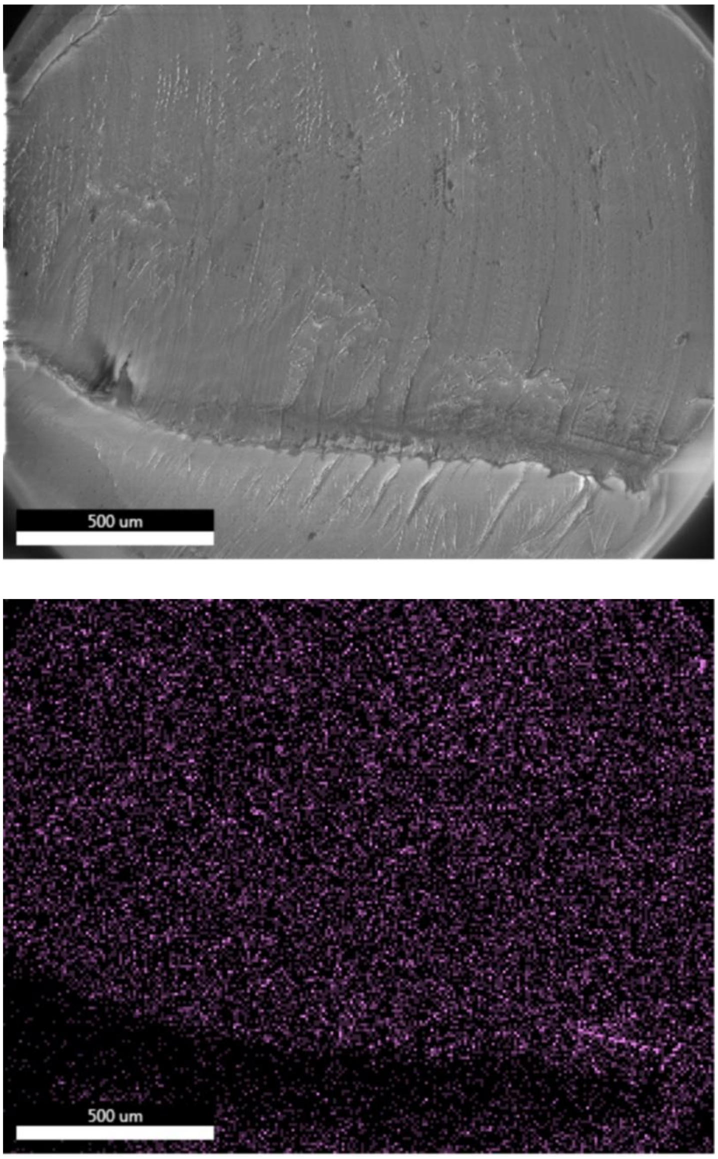


Figure S24: 0.1% $iBu_7$-SSQ-$NH_2$/PE

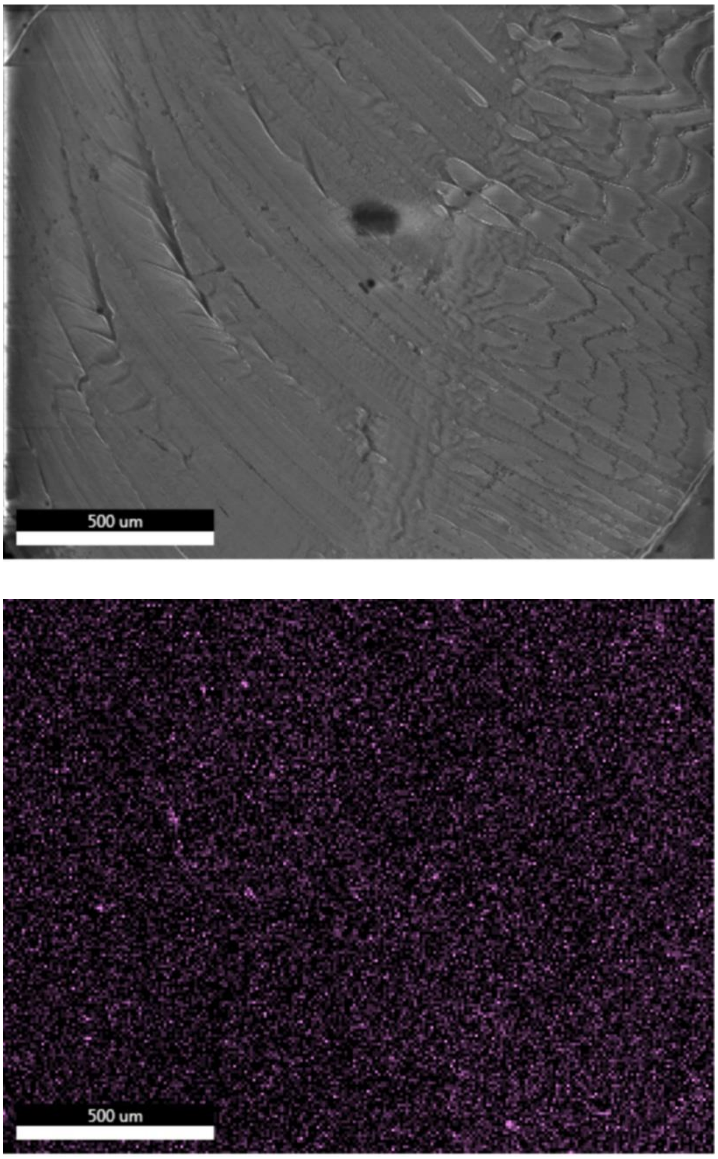


Figure S25: 0.5% $iBu_7$-SSQ-$NH_2$/PE

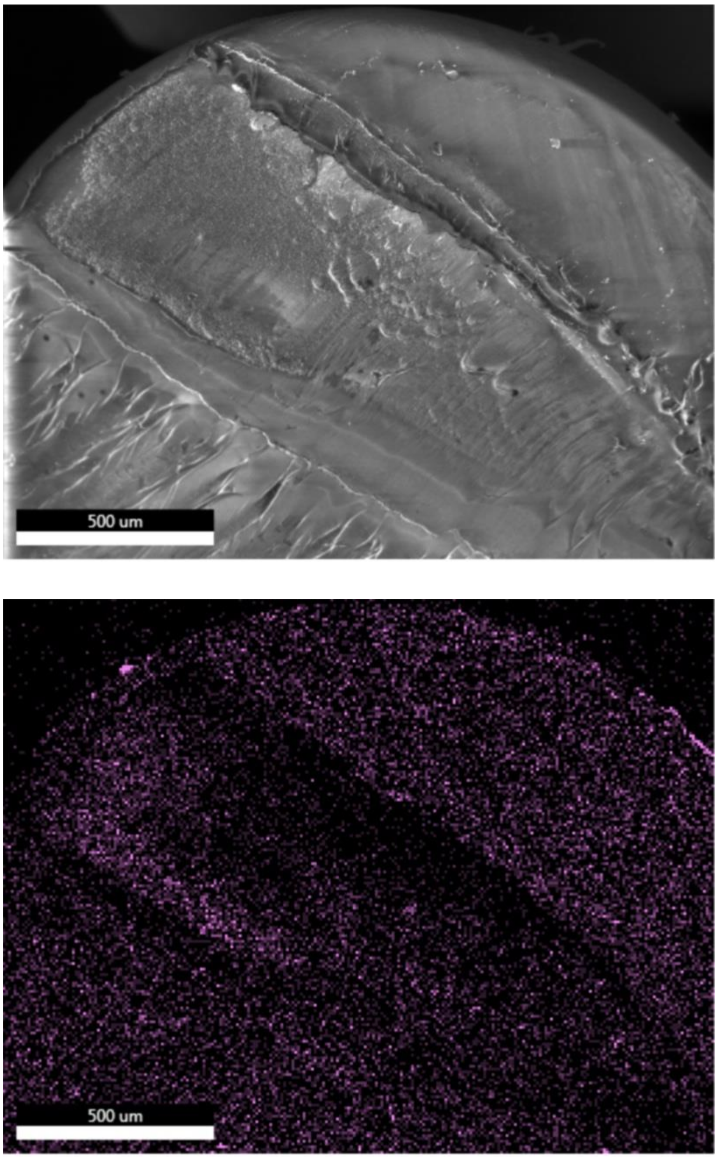


Figure S26: 1% $iBu_7$-SSQ-$NH_2$/PE

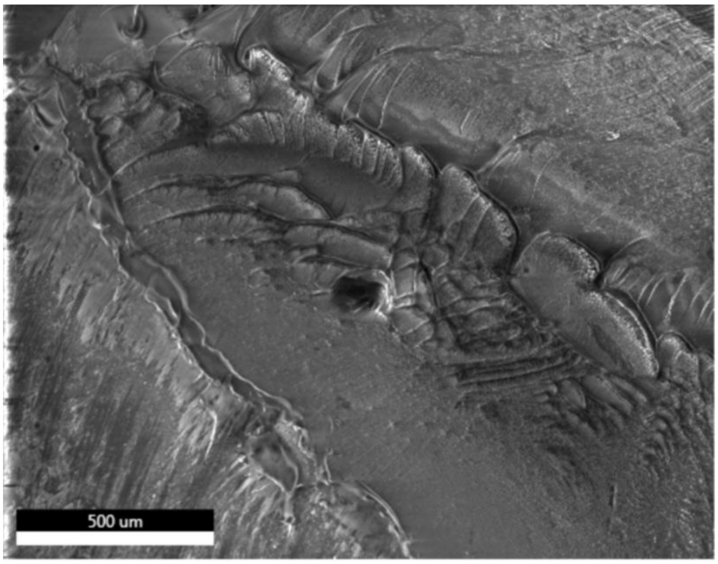


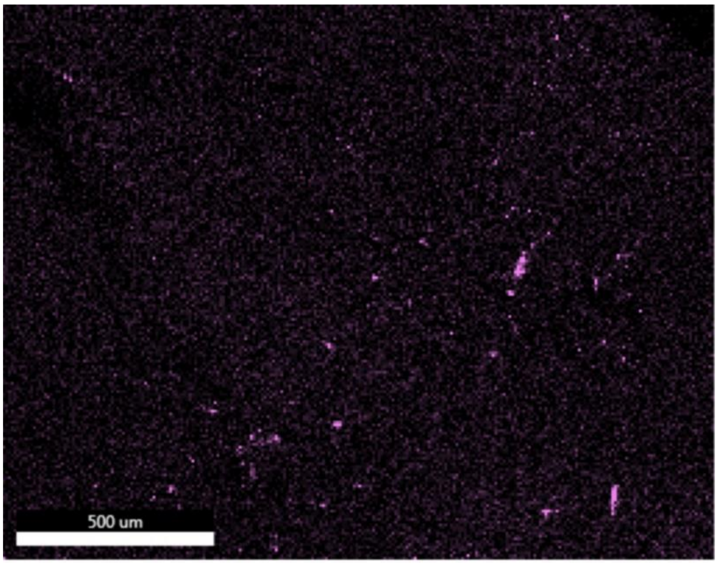


Figure S27: 0.5% $iBu_7$-SSQ-Vi/PE

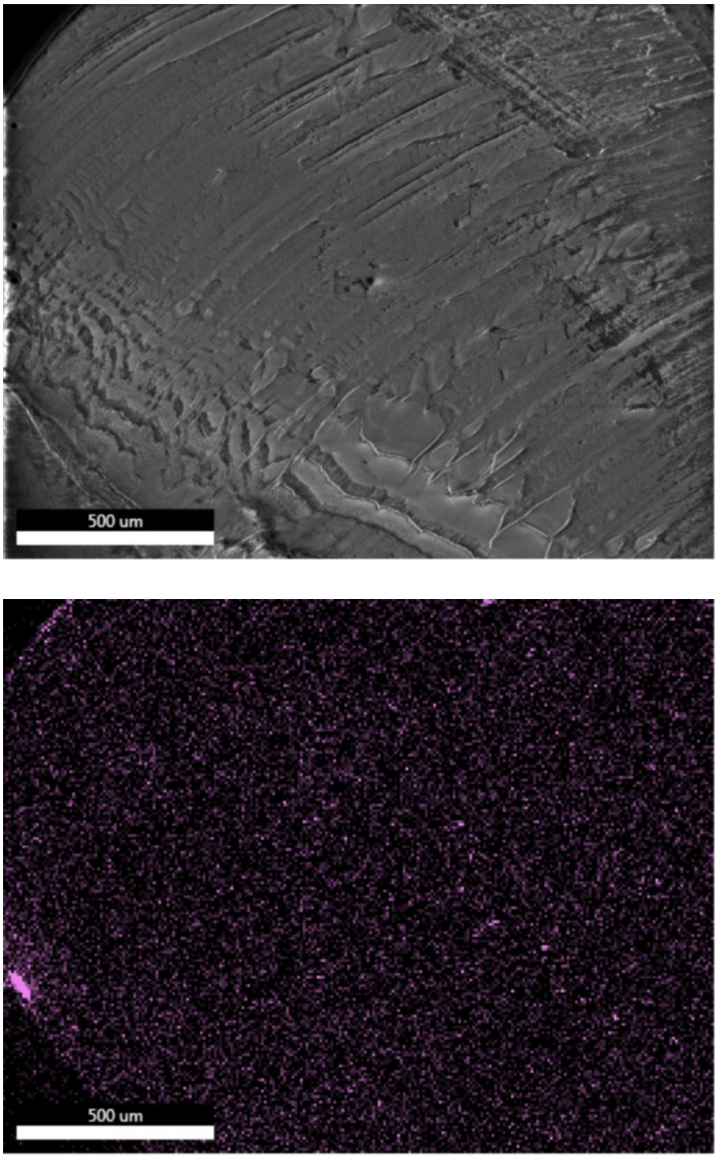


Figure S28: 1% $iBu_7$-SSQ-Vi/PE

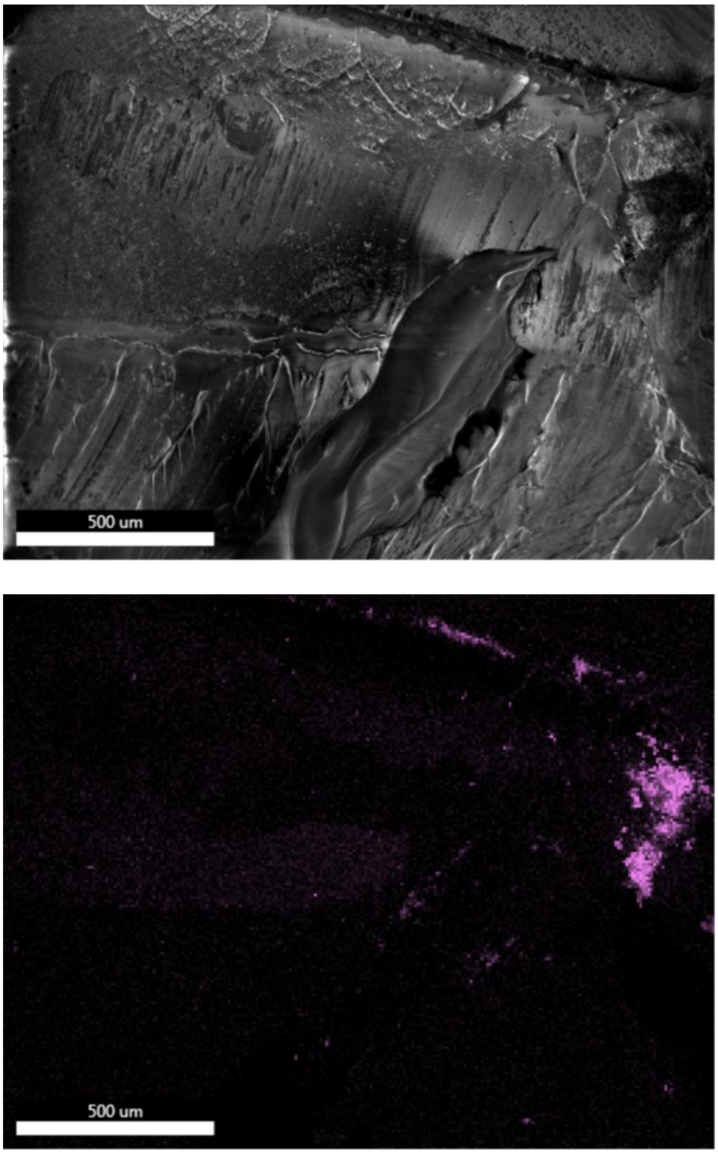


Figure S29: 1.5% $iBu_7$-SSQ-Vi/PE

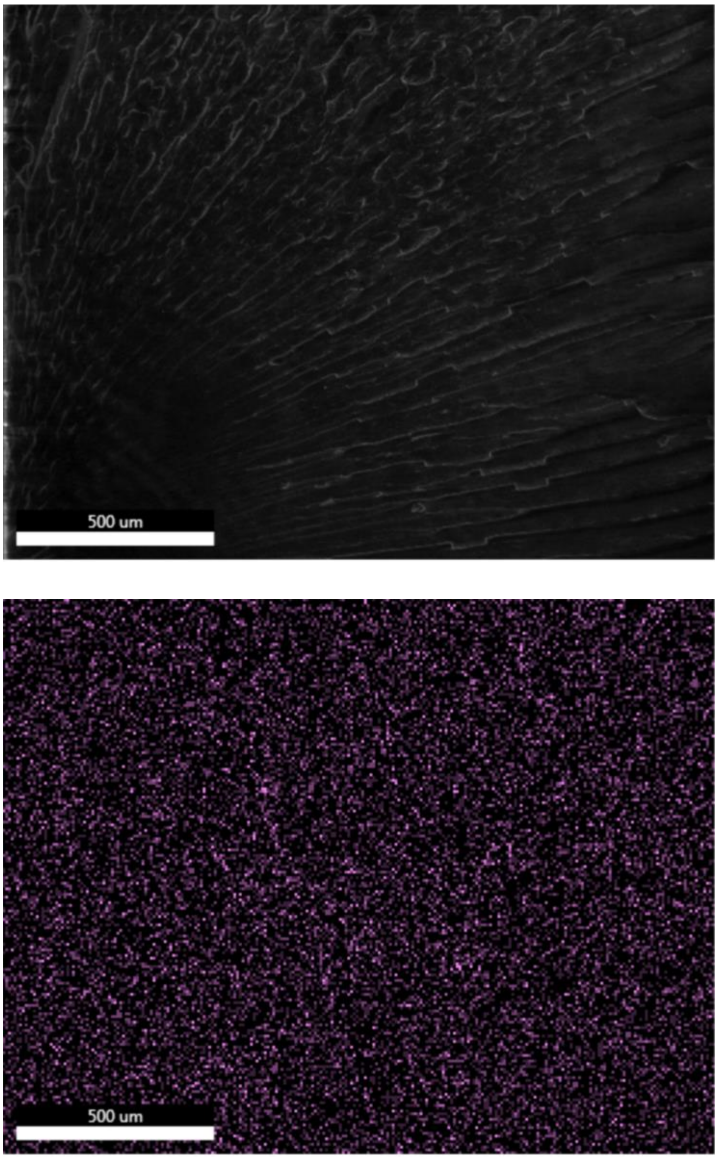


Figure S30: 0.5% $iBu_7$-SSQ-3OH/PE

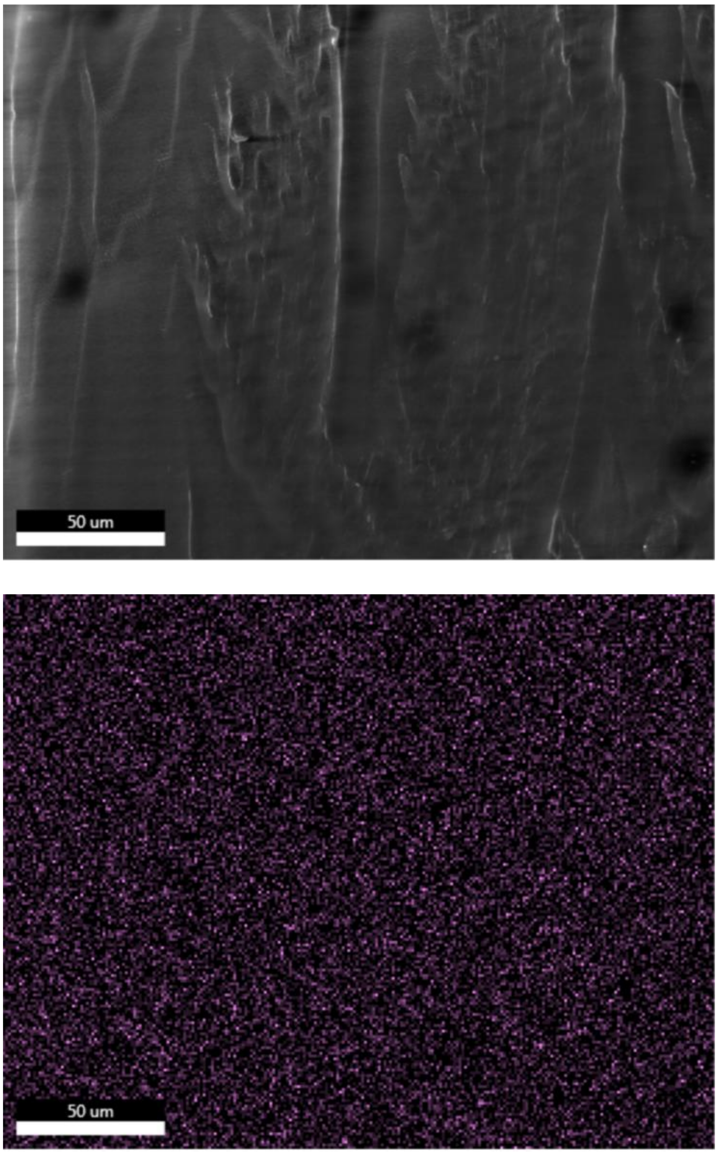


Figure S31: 0.5% $iBu_7$-SSQ-3OH/PE (higher magnification)

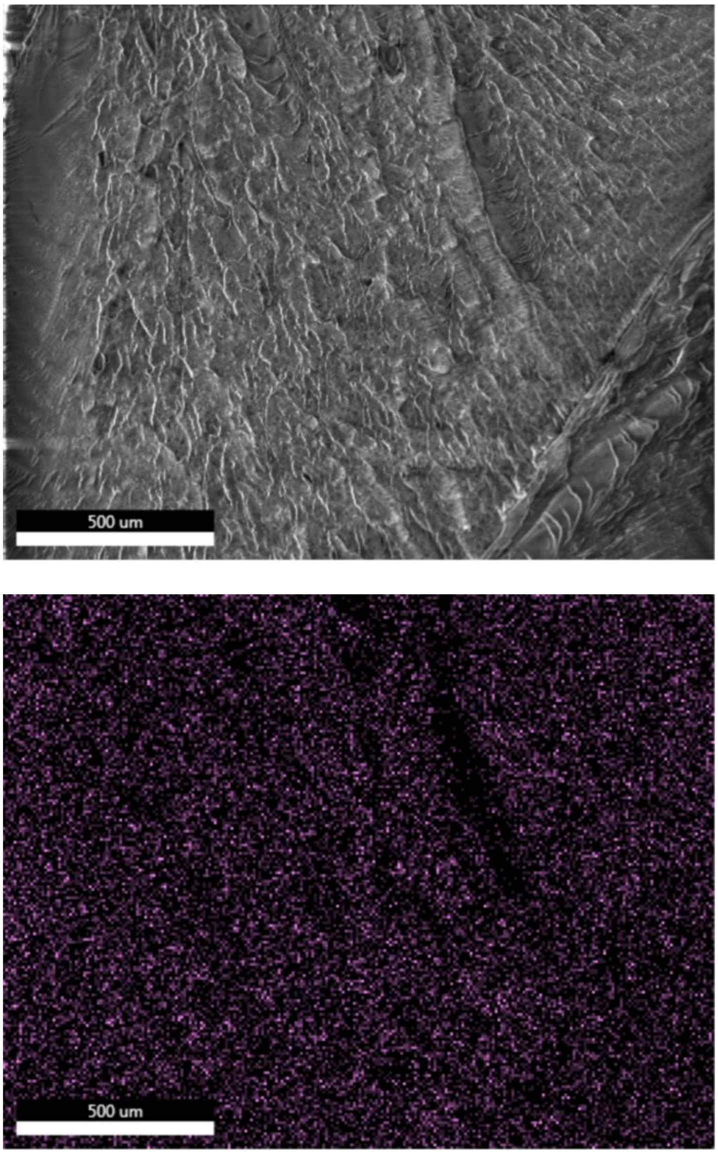


Figure S32: 1% $iBu_7$-SSQ-3OH/PE

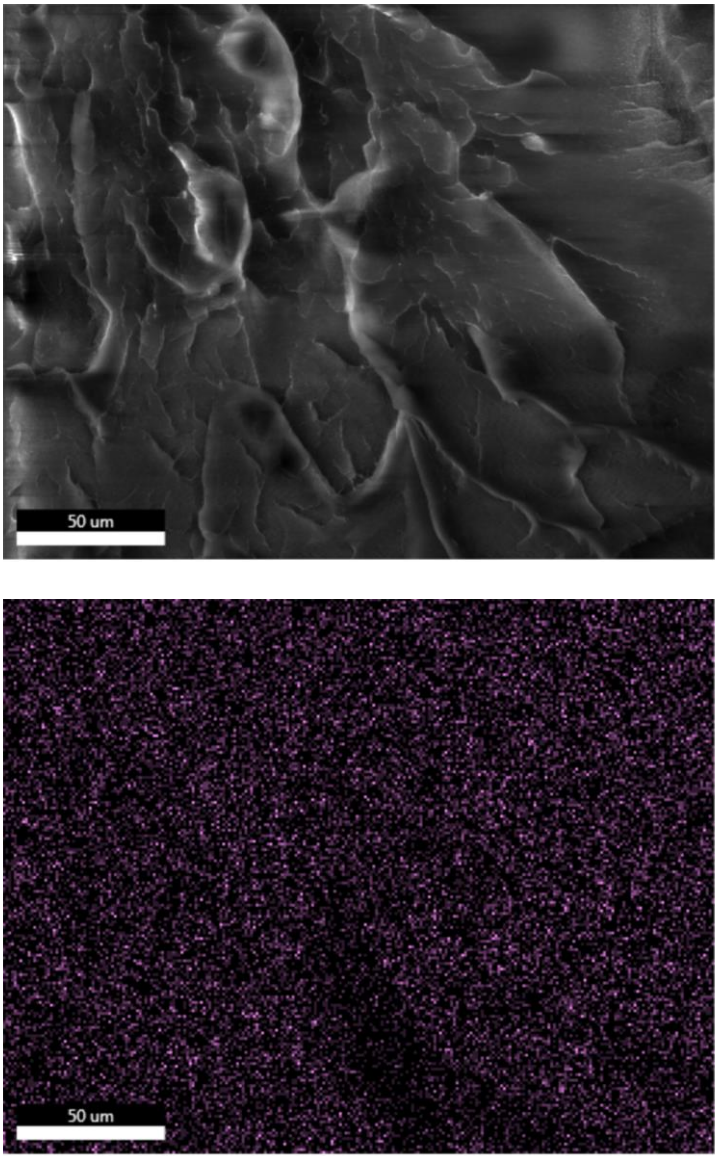


Figure S33: 1% $iBu_7$-SSQ-3OH (higher magnification)

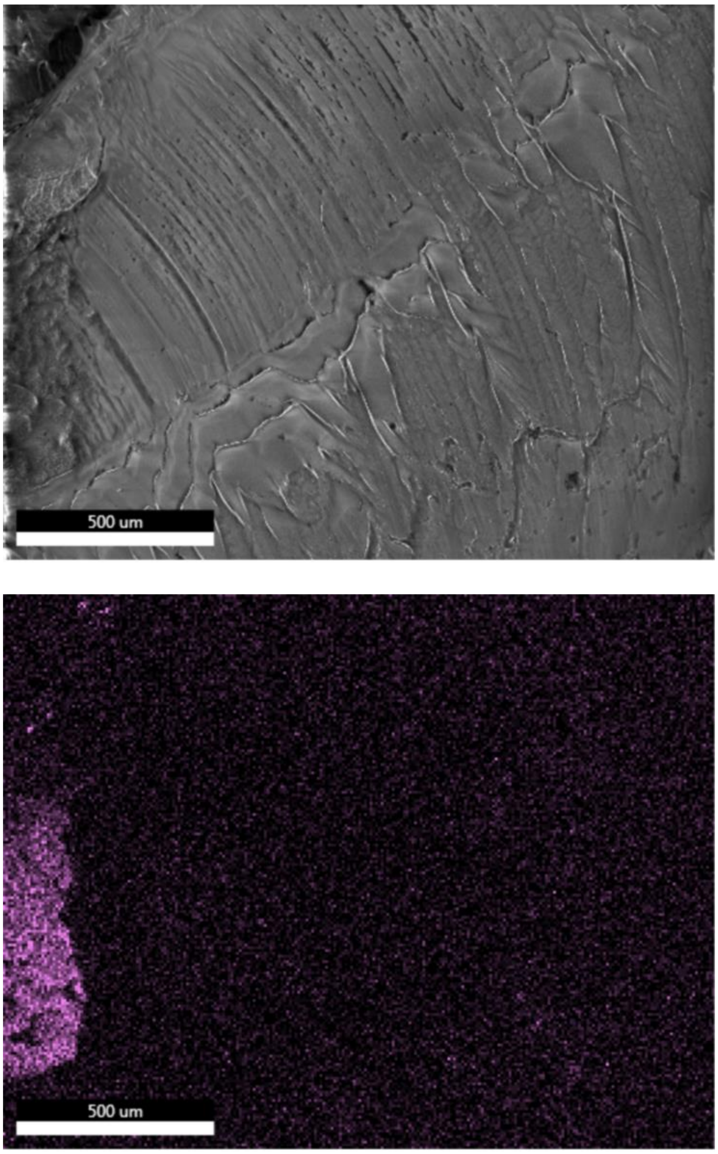


Figure S34: 1.5% $iBu_7$-SSQ-3OH

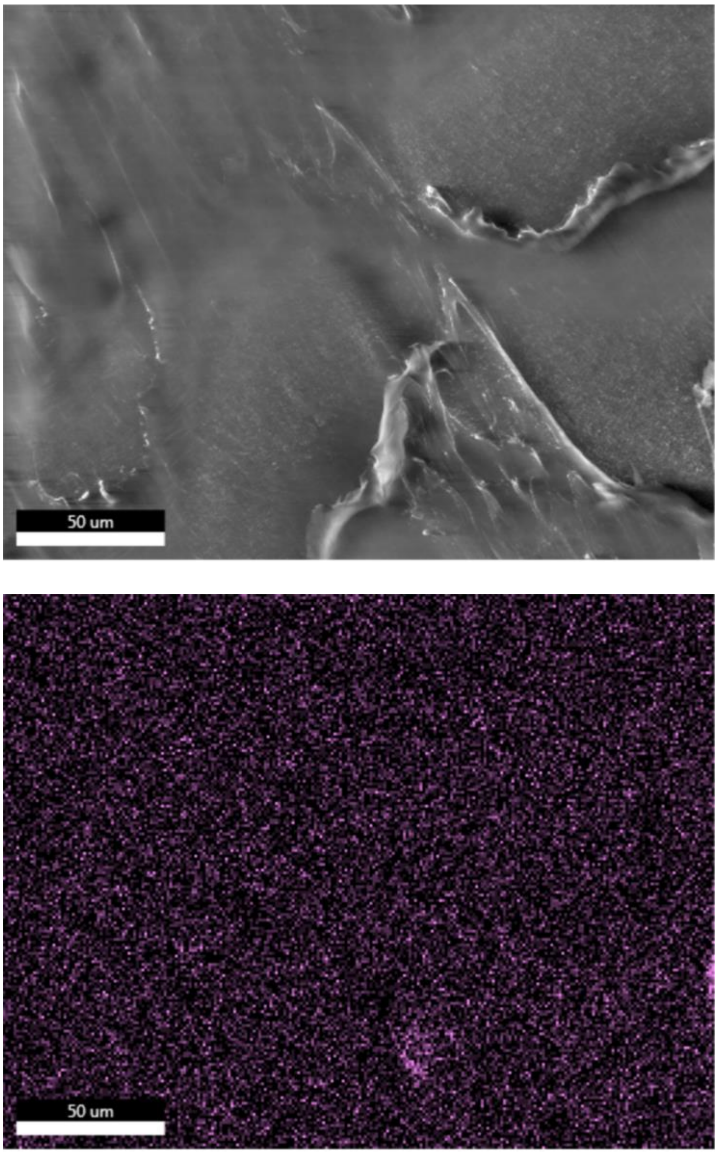


Figure S35: 1.5% $iBu_7$-SSQ-3OH (higher magnification)

## 5. Data Tables

**Table S2.** Crystallinity indices (CI) of the obtained composites measured by DSC.

| Additive | Concentration of additive [%] | | | |
|---|---|---|---|---|
| | 0.1 | 0.5 | 1.0 | 1.5 |
| | Crystallinity Index (CI) [%] | | | |
| Neat PE | 44.1 | | | |
| SSQ-8Cl | 45.8 | 47.1 | 45.5 | - |
| *i*$Bu_7$SSQ-Cl | 44.4 | 45.0 | 44.7 | - |
| *i*$Bu_7$SSQ-$NH_2$ | 44.5 | 46.3 | 44.0 | - |
| *i*$Bu_7$SSQ-Vi | - | 46.2 | 43.9 | 46.3 |
| *i*$Bu_7$SSQ-3OH | - | 47.1 | 46.6 | 46.5 |

**Table S3.** Heat Deflection Temperatures of the obtained composites.

| Additive | Concentration of additive [%] | | | |
|---|---|---|---|---|
| | 0.1 | 0.5 | 1.0 | 1.5 |
| | Heat Deflection Temperature [°C] | | | |
| Neat PE | 36.5 | | | |
| SSQ-8Cl | 36,9 | 36,4 | 35,7 | - |
| *i*$Bu_7$SSQ-Cl | 36,2 | 35,3 | 35,4 | - |
| *i*$Bu_7$SSQ-$NH_2$ | 35,2 | 35,4 | 35,6 | - |
| *i*$Bu_7$SSQ-Vi | - | 35,5 | 35,9 | 36.8 |
| *i*$Bu_7$SSQ-3OH | - | 37,2 | 36,6 | 36.2 |
